# Supplementary material for: Mechanisms and physiological function of daily haemoglobin oxidation rhythms in red blood cells
Source: EMBO J. 2023 Aug 9;42(19):e114164. doi: 10.15252/embj.2023114164 (PMC10548169; doi:10.15252/embj.2023114164)
Supplement: Supplementary file 1 — Expanded View Figures PDF [file EMBJ-42-e114164-s004.pdf]

Expanded View Figures

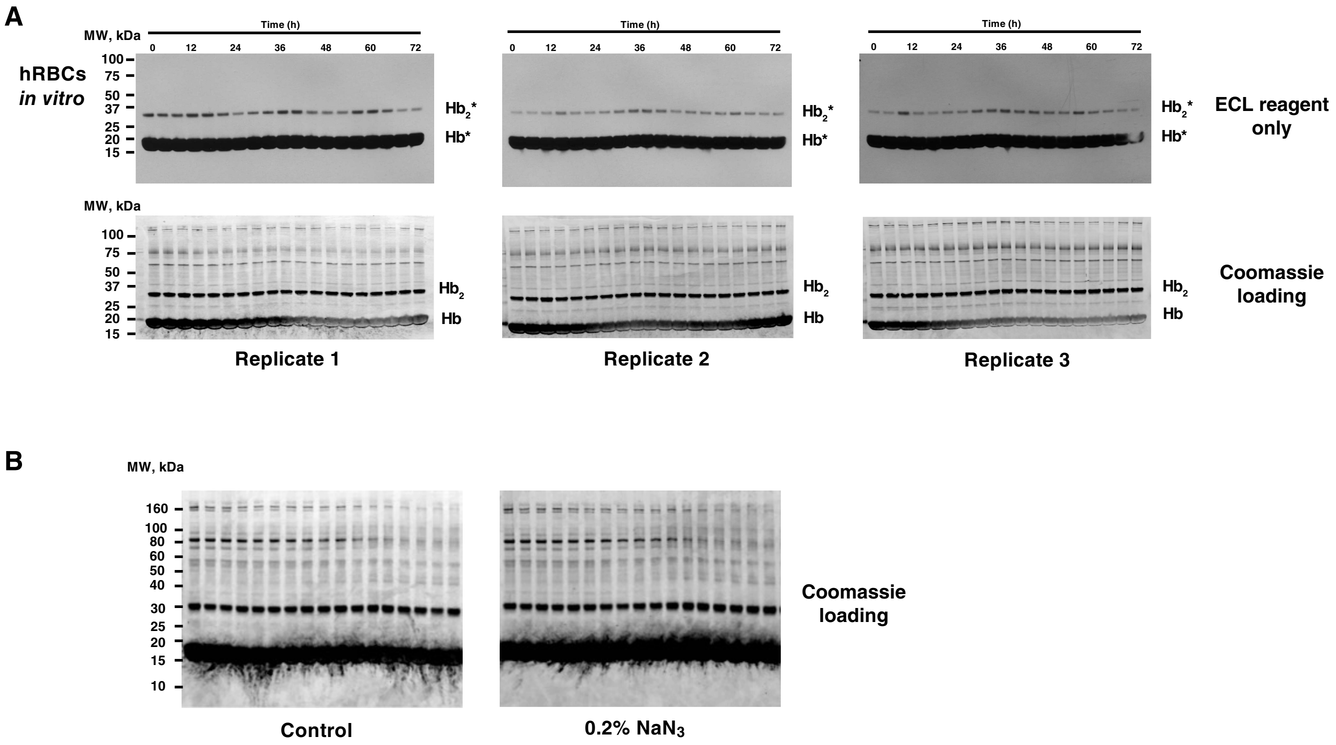

Figure EV1. Relating to Fig 1—Daily variation in haem-mediated peroxidase activity in human RBC time-course extracts on nitrocellulose membranes following SDS–PAGE under denaturing conditions.

A Uncropped blots and gels from *in vitro* human RBC time-course extracts are shown in Fig 1. Top panels show the chemiluminescence signal arising when nitrocellulose membranes were immediately incubated with ECL reagent, following SDS–PAGE and transfer onto nitrocellulose membranes. Please note that chemiluminescence is observed at molecular weights corresponding to Hb and Hb_2 without any antibody incubation or external source of peroxidase activity. For practical reasons, the Hb_2* band was used for quantification presented in Fig 1 since Hb* quickly saturated the X-ray film used to detect chemiluminescence. Bottom panels show associated Coomassie-stained gels (loading control).

B Uncropped coomassie-stained gels from *in vitro* human RBC time-course extracts shown in Fig 1B. In Fig 1B, before incubation with ECL reagent, membranes were incubated for 30 min in PBS ± 0.2% sodium azide.

© 2023 The Authors

A

chain A

```
1   VLSPADKTNV KAAWGKVGAH AGEYGAEALE RMFLSFPTTK TYFPHFDLSH
51  GSAQVKGHGK KVADALTNAV AHVDDMPNAL SALSDLHAHK LRVDPVNFKL
101 LSHCLLVTLA AHLPAEFTPA VHASLDKFLA SVSTVLTSKY
```

chain B

```
1   VHLTPEEKSA VTALWGKVNV DEVGGEALGR LLVVYPWTQR FFESFGDLST
51  PDAVMGNPKV KAHGKKVLGA FSDGLAHLDN LKGTFATLSE LHCDKLHVDP
101 ENFRLLGNVL VCVLAHHFGK EFTPPVQAAY QKVVAGVANA LAHKY
```

B

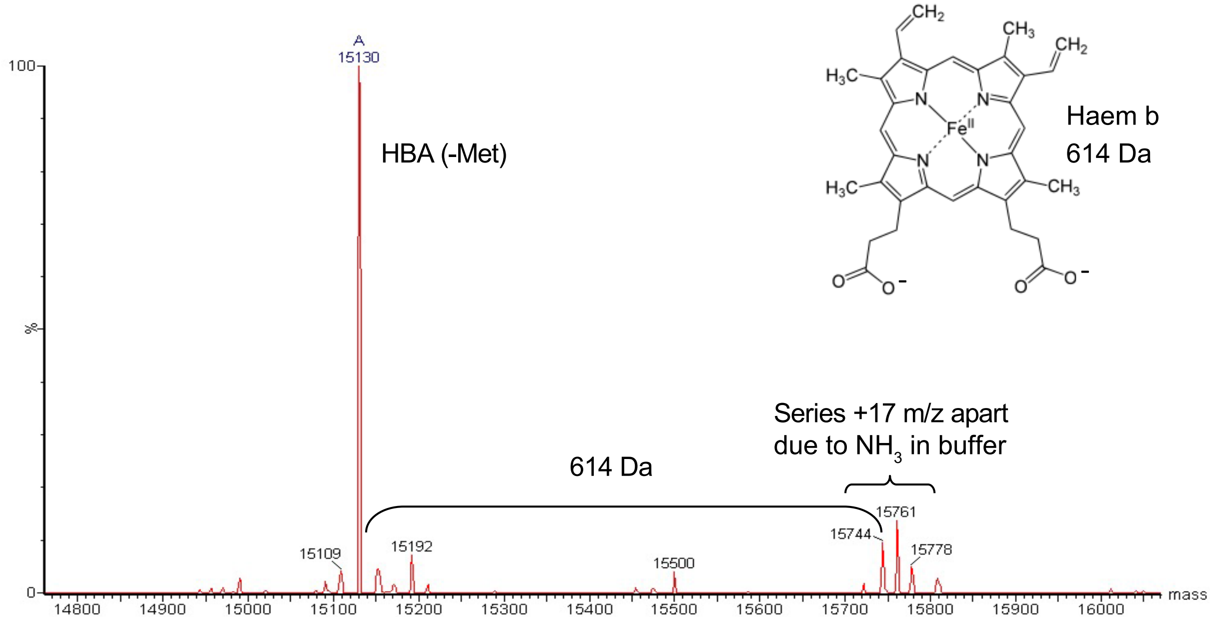

Figure EV2. Relating to Fig 1—Mass spectrometry of Ni-NTA-purified peroxidase band.

A Mass spectrometry of tryptic digests of the putative haem-Hb species purified by Ni-NTA affinity chromatography under denaturing conditions (Fig 1C) revealed Hb chains A and B (HBA/HBB) as the major species with very high primary sequence coverage (indicated in red).

B The haem-Hb species purified by Ni-NTA affinity chromatography under denaturing conditions was excised and the undigested polypeptide analysed by mass spectrometry. Raw mass spectrometry data showing signal at the expected molecular weight of HBA protein are shown, with additional peaks at +614 Da—the mass of haem b (insert).

© 2023 The Authors

Figure EV3. Relating to Figs 2 and 3—Circadian variation in PRX-$SO_{2/3}$ and haem-mediated peroxidase activity in mouse RBC in vitro time-course extracts (A–C) on nitrocellulose membranes after denaturing SDS–PAGE transfer and human RBC in vivo time-course extracts (D).

A Top panels show uncropped representative PRX-$SO_{2/3}$ immunoblots of RBC time-course extracts from each genotype shown in Fig 2. Middle panels show associated Coomassie-stained gels (loading control) where Hb is the major protein. The previously reported occurrence of cross-linked Hb dimers (Hb_2) is readily observable at ~32 kDa, and the identity of the band marked as Hb_2 was confirmed by mass spectrometry (not shown). Bottom three panels show the chemiluminescence signal arising when replicate nitrocellulose membranes were immediately incubated with ECL reagent, following transfer from SDS–PAGE. Please note that chemiluminescence is observed at molecular weights corresponding to Hb and Hb_2 without any antibody incubation or external source of peroxidase activity. For practical reasons, the Hb_2* band was used for quantification presented in Fig 2, since Hb* quickly saturated the X-ray film used to detect chemiluminescence (as shown in C, below).

B The intrinsic chemiluminescence from bands at molecular weights corresponding to Hb and Hb_2, as well as free haem, was first observed faintly as non-specific bands in overexposed PRX-$SO_{2/3}$ immunoblots.

C Further investigation revealed that this peroxidase activity was not attributable to non-specific antibody binding, since it is readily observed in RBC extracts upon addition of ECL reagents to nitrocellulose membranes, immediately after transfer from SDS–PAGE. Please note the very high activity of Hb* compared with Hb_2*, which is consistent with the relative levels of Hb and Hb_2 detected by Coomassie in (A). Also, note the signal due to free haem that is apparent upon longer exposures (right). Interestingly, compared with human RBC time courses (O'Neill & Reddy, 2011; Henslee *et al*, 2017), we observed that murine PRX-$SO_{2/3}$ immunoreactivity was extremely high during the first 24 h of each 72-h time course (Fig EV3A). We attribute this to the different conditions under which blood was collected: blood was collected from mice culled by CO_2 asphyxiation during their habitual rest phase by cardiac puncture and exposed immediately to atmospheric oxygen levels, whereas human blood was collected from subjects during their habitual active phase through venous collection into a vacuum-sealed collection vial. Thus, the initial high PRX-$SO_{2/3}$ signal in mice may be related to CO_2 acidification of the blood during culling, which affects PRX-$SO_{2/3}$ but does not affect Hb oxidation status.

D Uncropped blots and gels from human blood time course sampled *in vivo* are shown in Fig 3. Top panels show the chemiluminescence signal arising when nitrocellulose membranes were immediately incubated with ECL reagent, following SDS–PAGE and transfer onto nitrocellulose membranes. Please note that chemiluminescence is observed at molecular weights corresponding to Hb and Hb_2 without any antibody incubation or external source of peroxidase activity. For practical reasons, the Hb_2* band was used for quantification presented in Fig 3, since Hb* quickly saturated the X-ray film used to detect chemiluminescence. Bottom panels show associated Coomassie-stained gels (loading control).

© 2023 The Authors

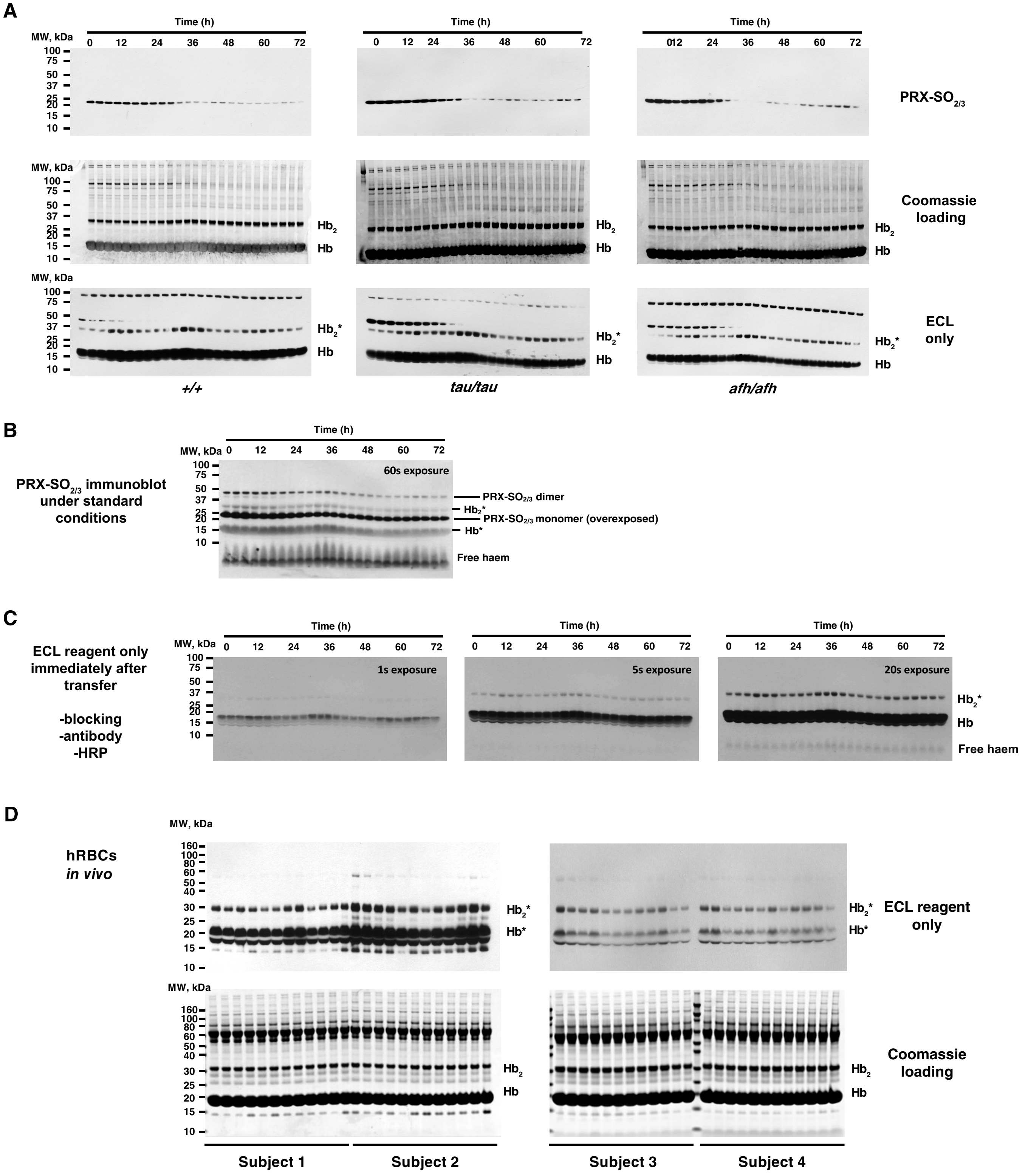

Figure EV3.

© 2023 The Authors

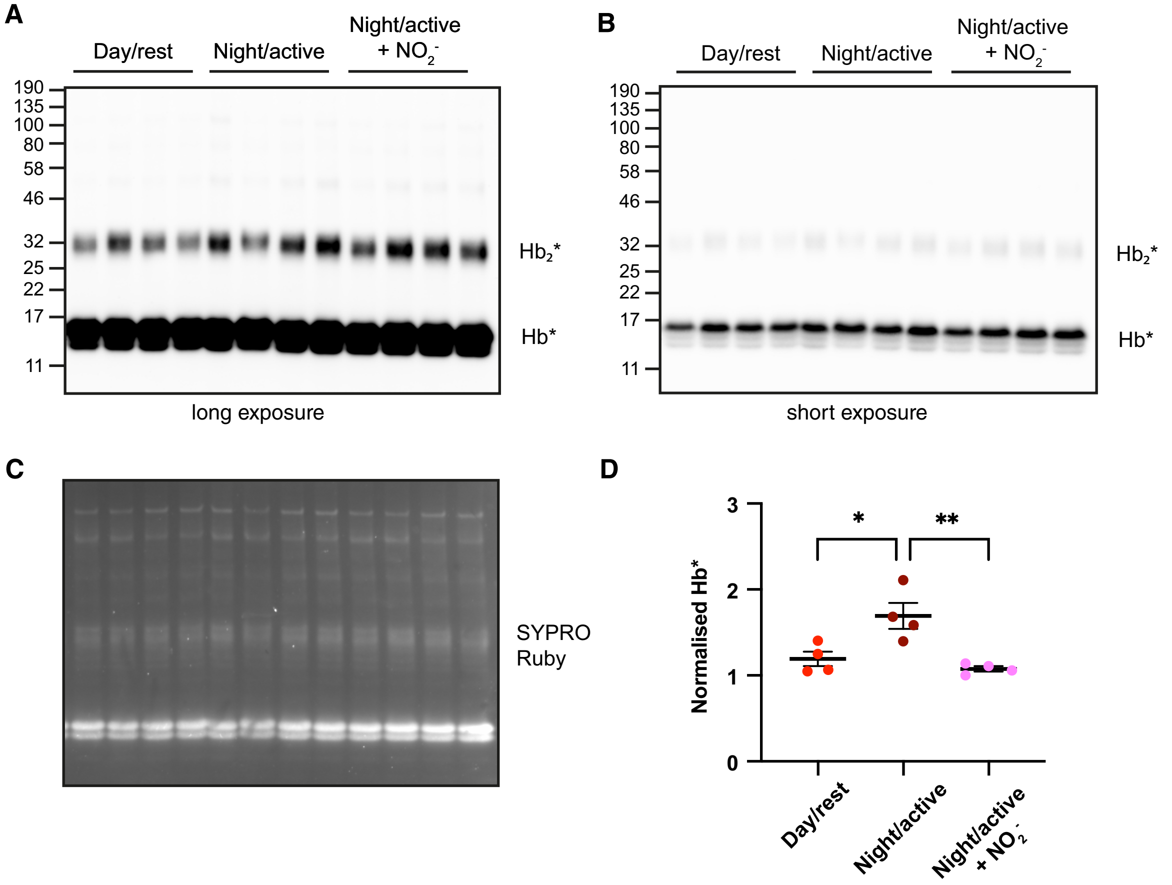

Figure EV4. Relating to Fig 4—Uncropped nitrocellulose membranes and gels as presented in Fig 4.

A Long exposure, for quantification of the Hb_2* band, as presented in Fig 4.
B Short exposure, showing Hb* activity.
C SYPRO Ruby uncropped gel. The lower band of SYPRO Ruby (corresponding to Hb monomer) is presented in Fig 4, but the intensity of the whole lane was used for quantification of loading.
D Quantification of Hb* band of from four mice in a short chemiluminescent exposure, using same methods as Fig 4. The same result is observed when quantifying Hb* as Hb_2*. One-way ANOVA, $P = 0.0027$; Sidak's multiple comparisons test displayed on graph.

Data information: In (D), data are presented as mean ± SEM with individual points. *$P \leq 0.05$, **$P \leq 0.01$.

© 2023 The Authors
