# Supplementary figures and images for: Mechanisms and physiological function of daily haemoglobin oxidation rhythms in red blood cells
Source: EMBO J. 2023 Aug 9;42(19):e114164. doi: 10.15252/embj.2023114164 (PMC10548169; doi:10.15252/embj.2023114164)

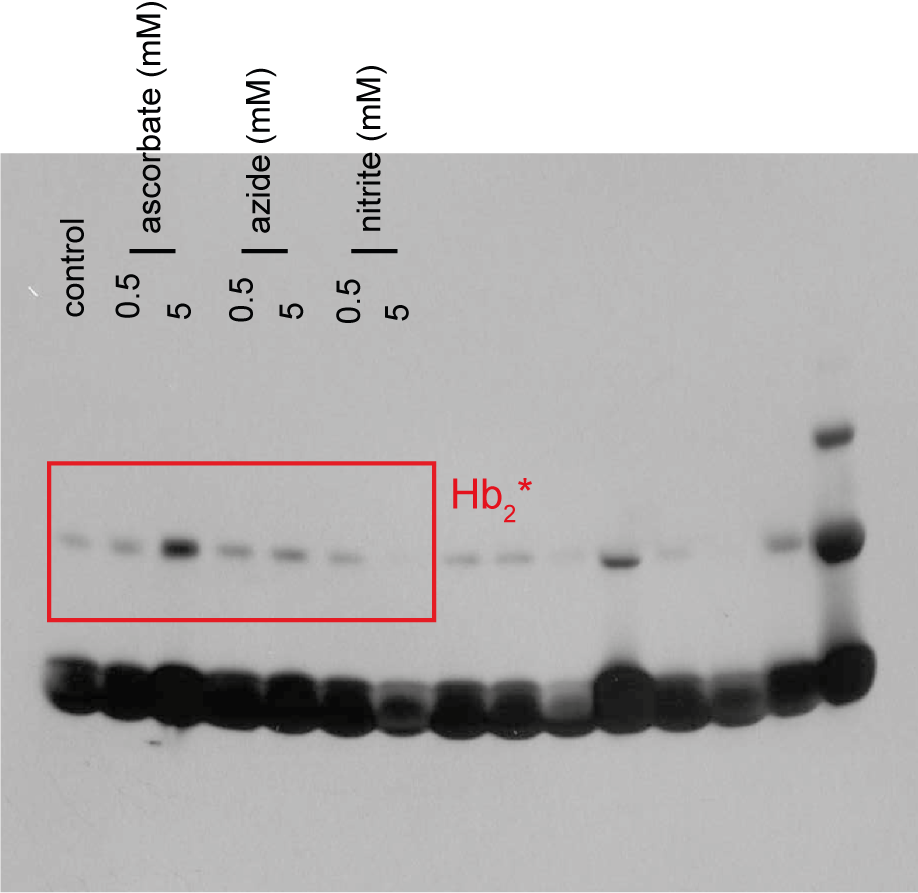

Supplement: Supplementary file 3 — Source Data for Figure 1 [file EMBJ-42-e114164-s003.zip › Figure 1/1H/ECL membrane for 1H.tif]

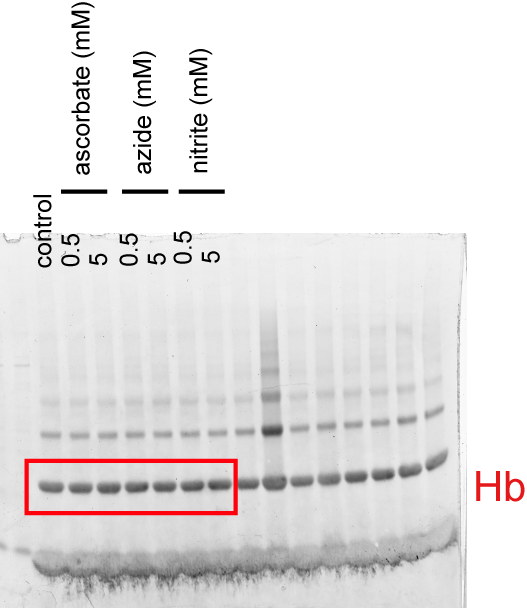

Supplement: Supplementary file 3 — Source Data for Figure 1 [file EMBJ-42-e114164-s003.zip › Figure 1/1H/coomassie gel for 1H.tif]

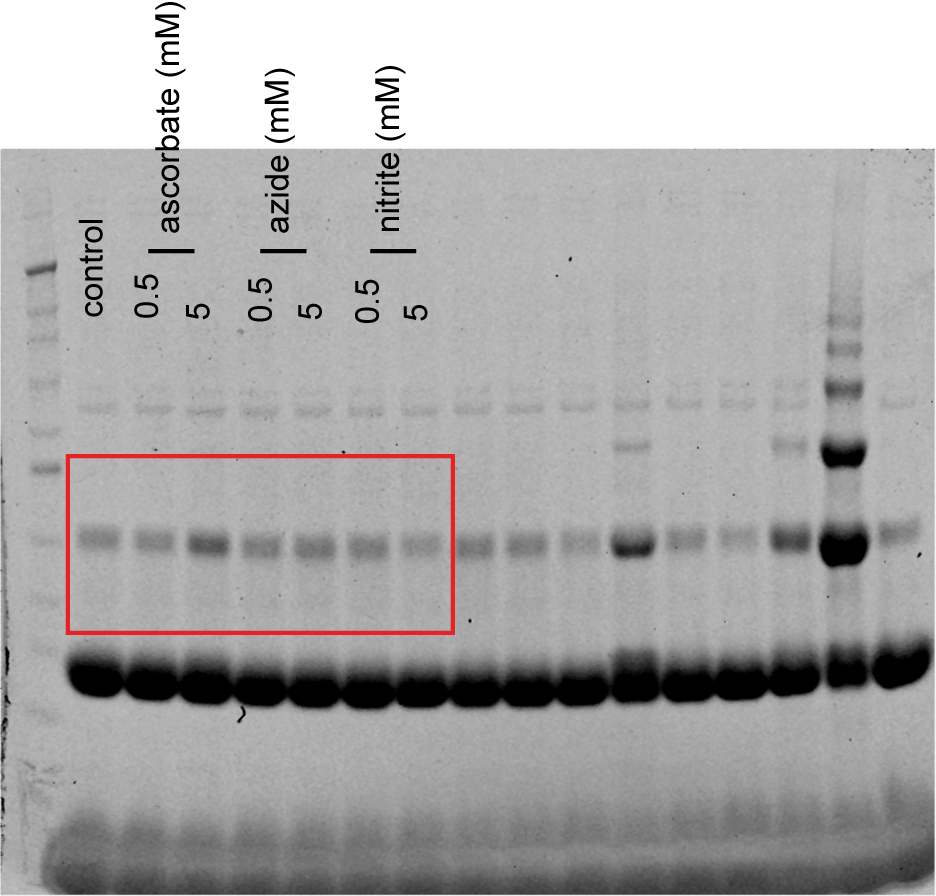

Supplement: Supplementary file 3 — Source Data for Figure 1 [file EMBJ-42-e114164-s003.zip › Figure 1/1H/in gel haem stain for 1H.tif]

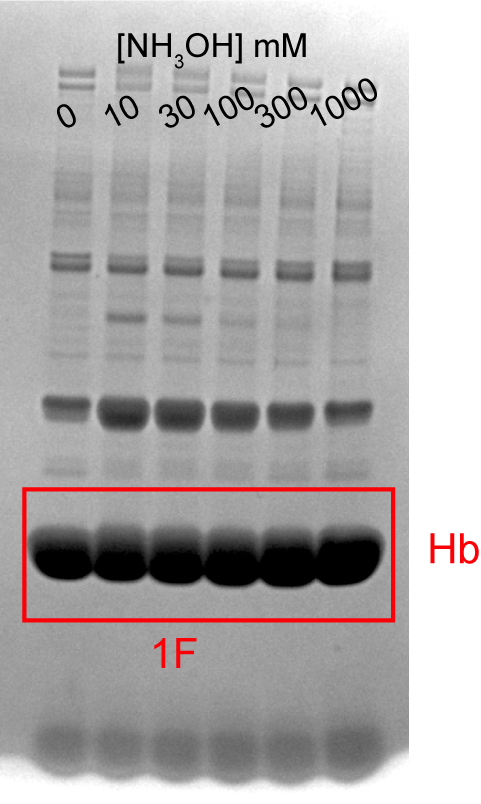

Supplement: Supplementary file 3 — Source Data for Figure 1 [file EMBJ-42-e114164-s003.zip › Figure 1/1F/coomassie gel for 1F.tif]

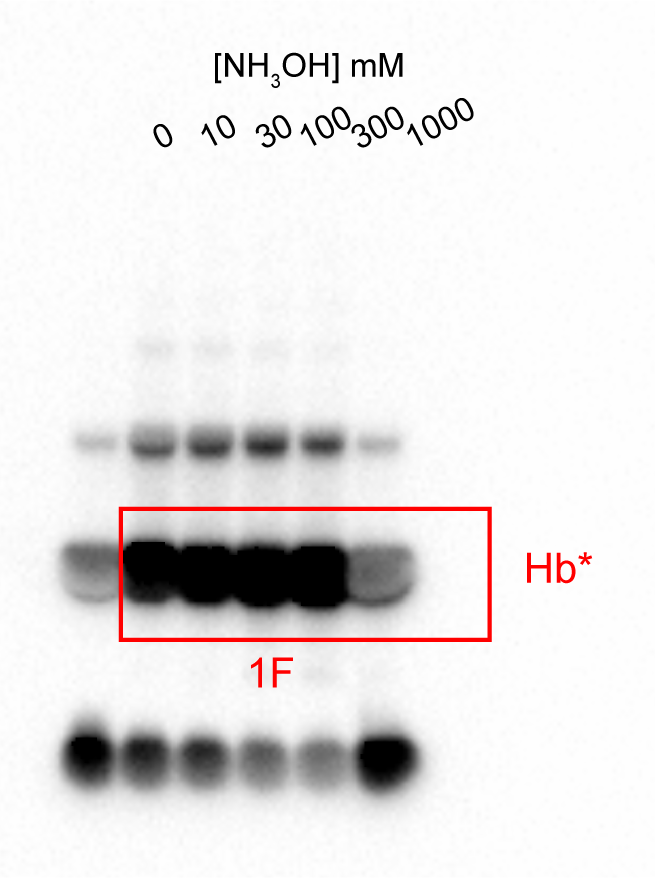

Supplement: Supplementary file 3 — Source Data for Figure 1 [file EMBJ-42-e114164-s003.zip › Figure 1/1F/ECL membrane for 1F.tif]

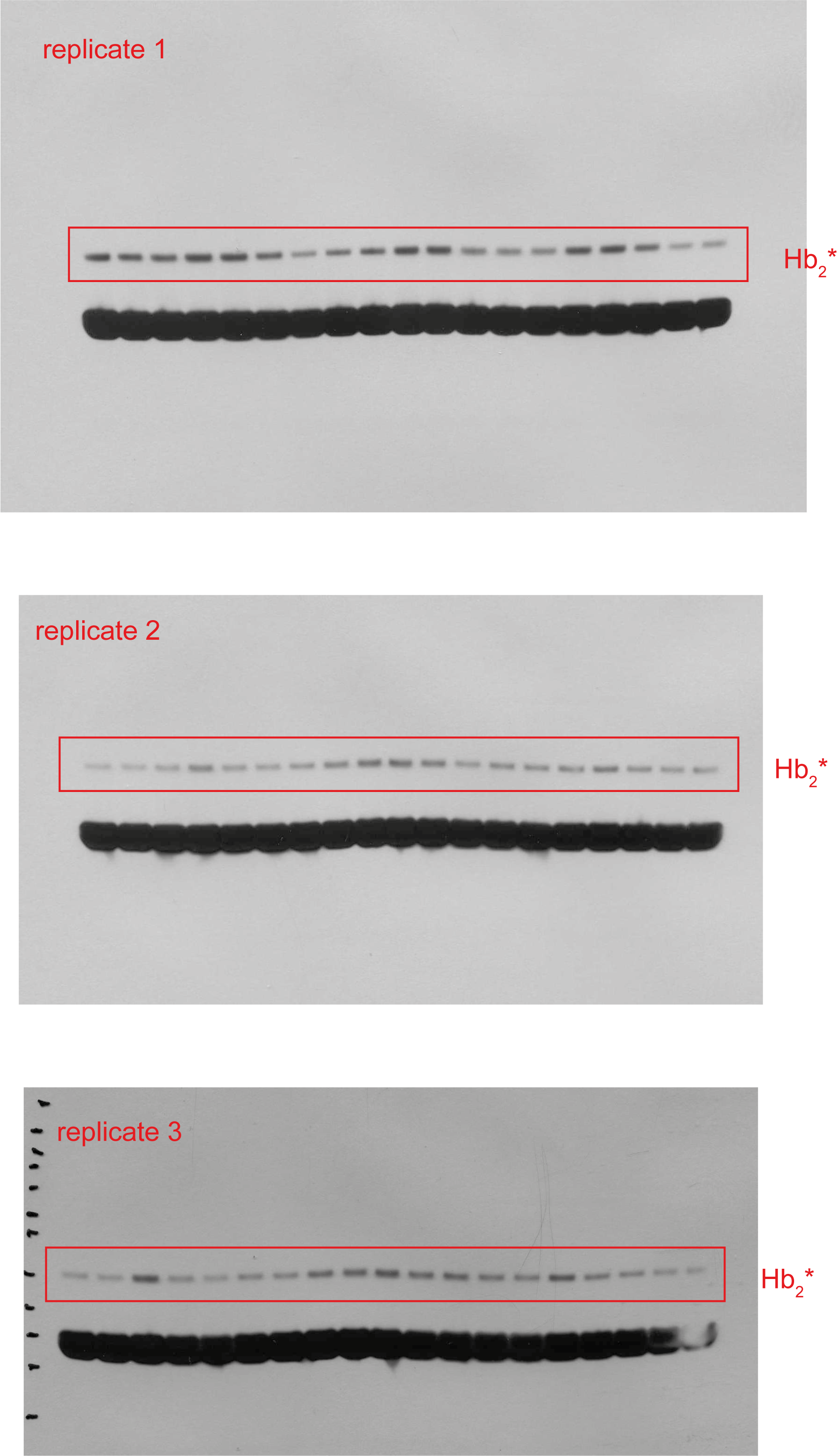

Supplement: Supplementary file 3 — Source Data for Figure 1 [file EMBJ-42-e114164-s003.zip › Figure 1/1A/ECL only membranes for three replicates.tif]

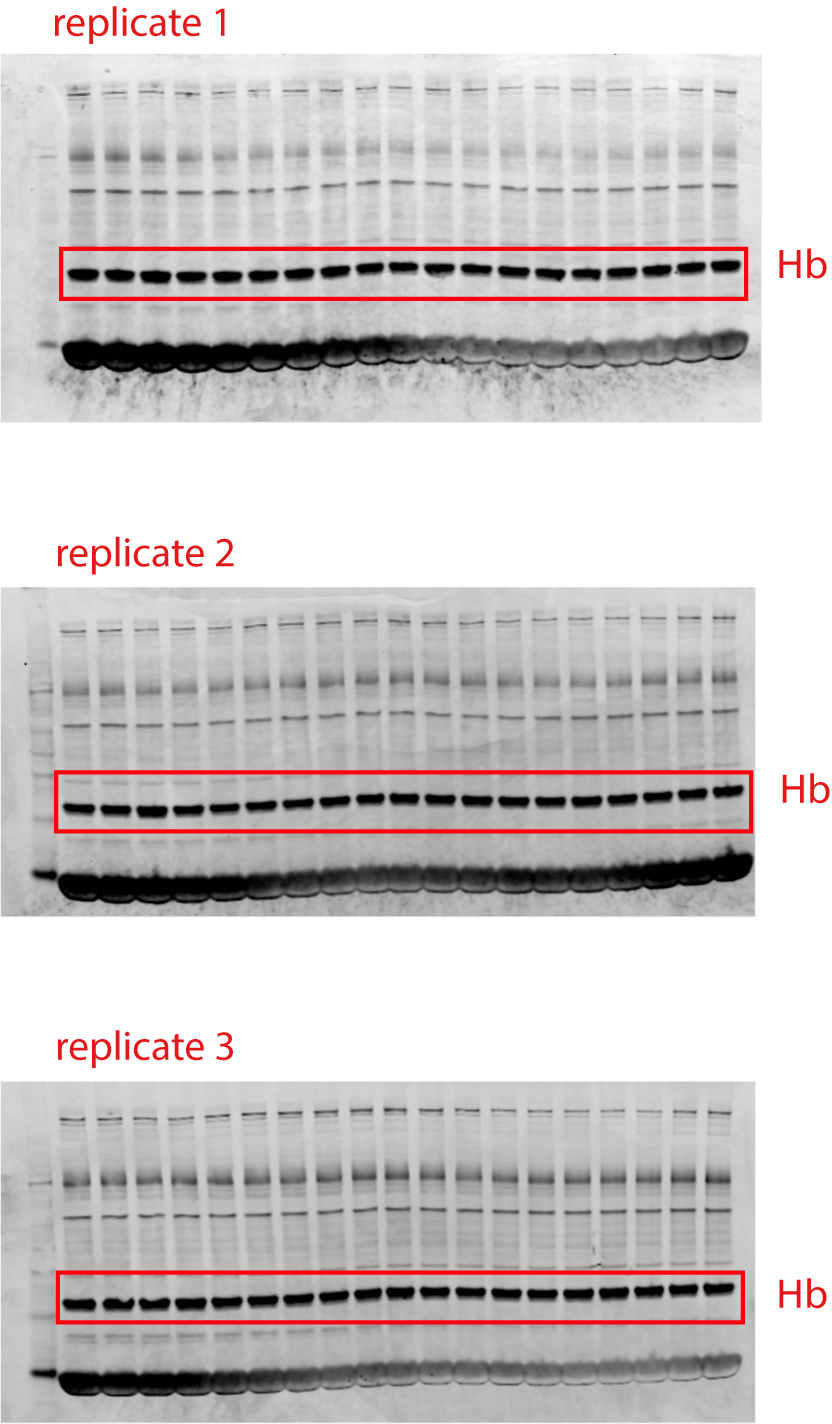

Supplement: Supplementary file 3 — Source Data for Figure 1 [file EMBJ-42-e114164-s003.zip › Figure 1/1A/coomassie gels for three replicates.tif]

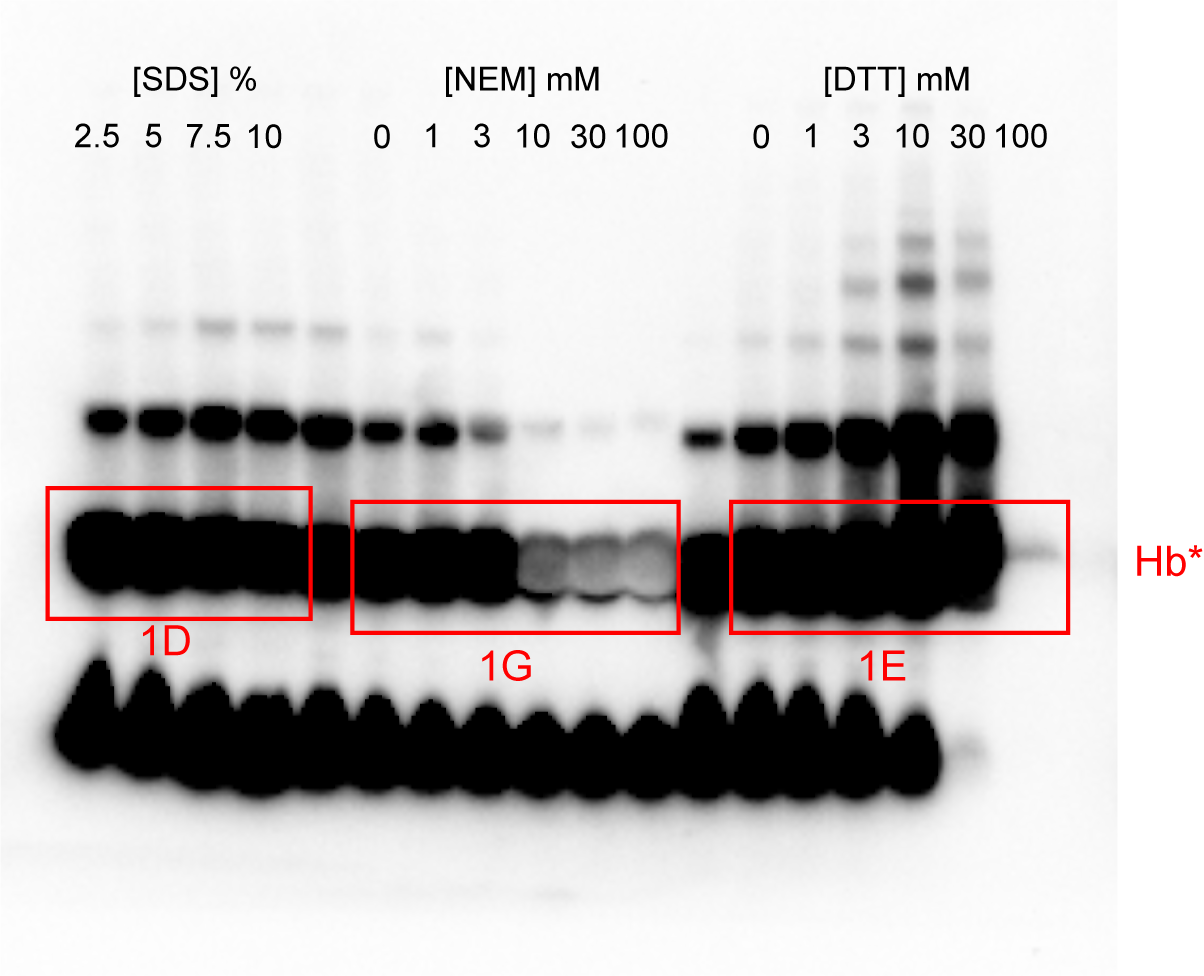

Supplement: Supplementary file 3 — Source Data for Figure 1 [file EMBJ-42-e114164-s003.zip › Figure 1/1D, E and G/ECL membrane for 1D, 1E and 1G.tif]

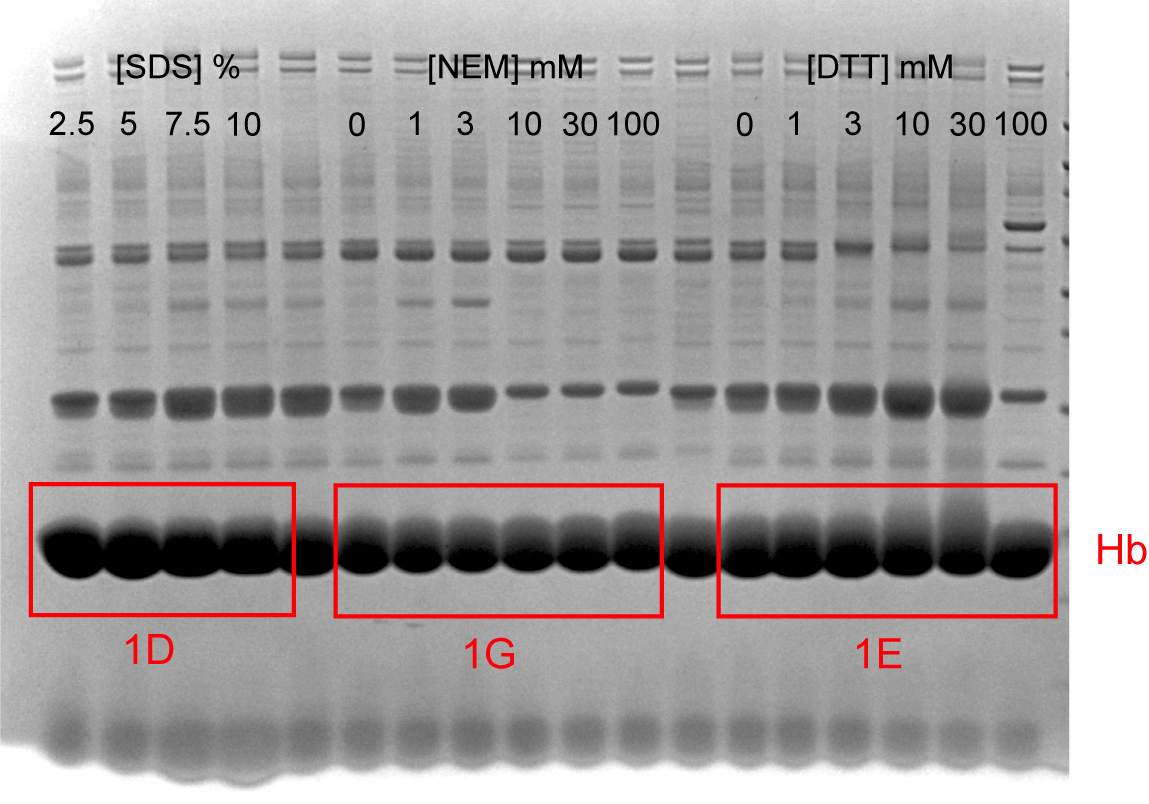

Supplement: Supplementary file 3 — Source Data for Figure 1 [file EMBJ-42-e114164-s003.zip › Figure 1/1D, E and G/coomassie gel for 1D, 1E and 1G.tif]

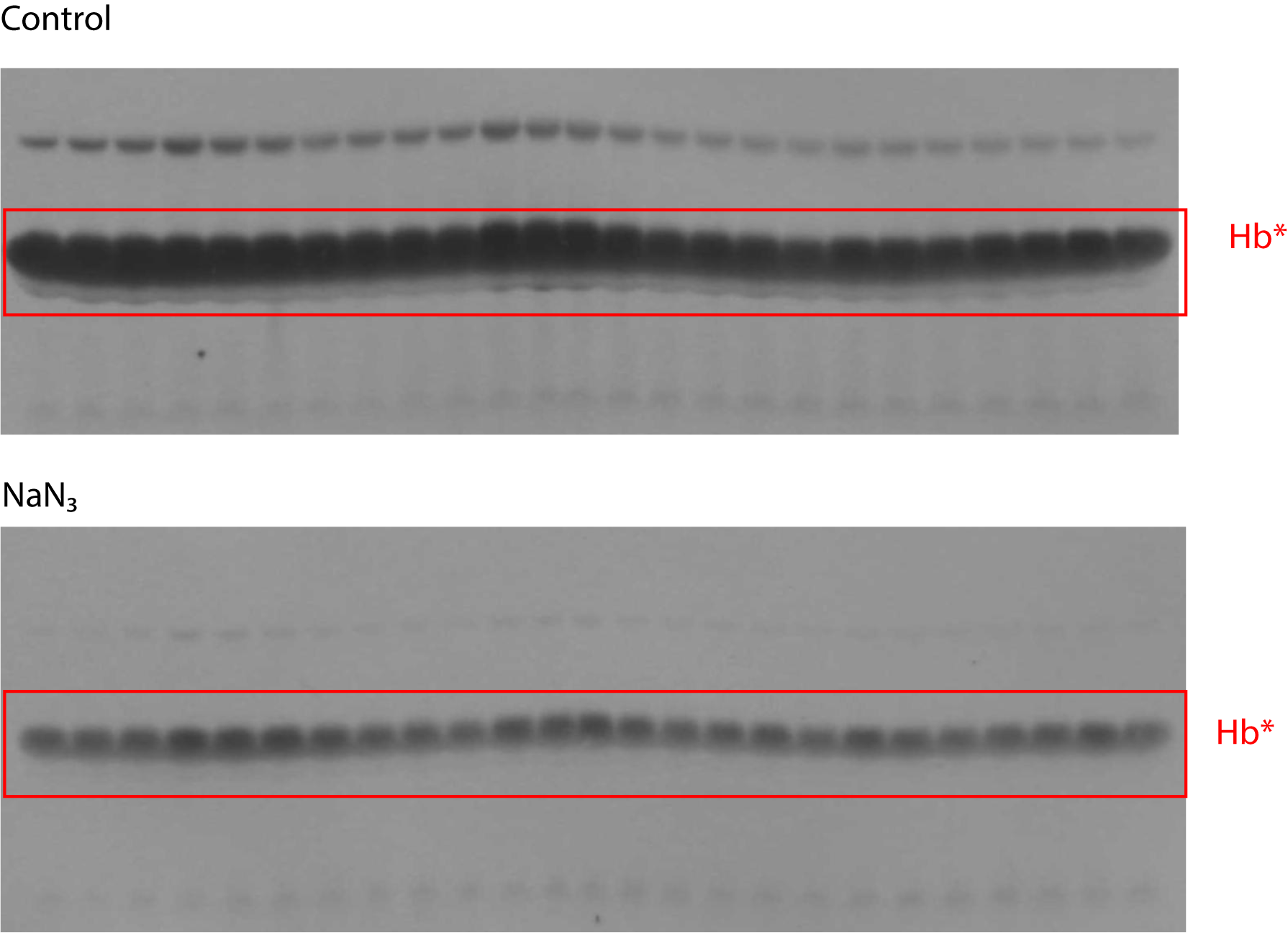

Supplement: Supplementary file 3 — Source Data for Figure 1 [file EMBJ-42-e114164-s003.zip › Figure 1/1B/ECL membrane control vs NaN3.tif]

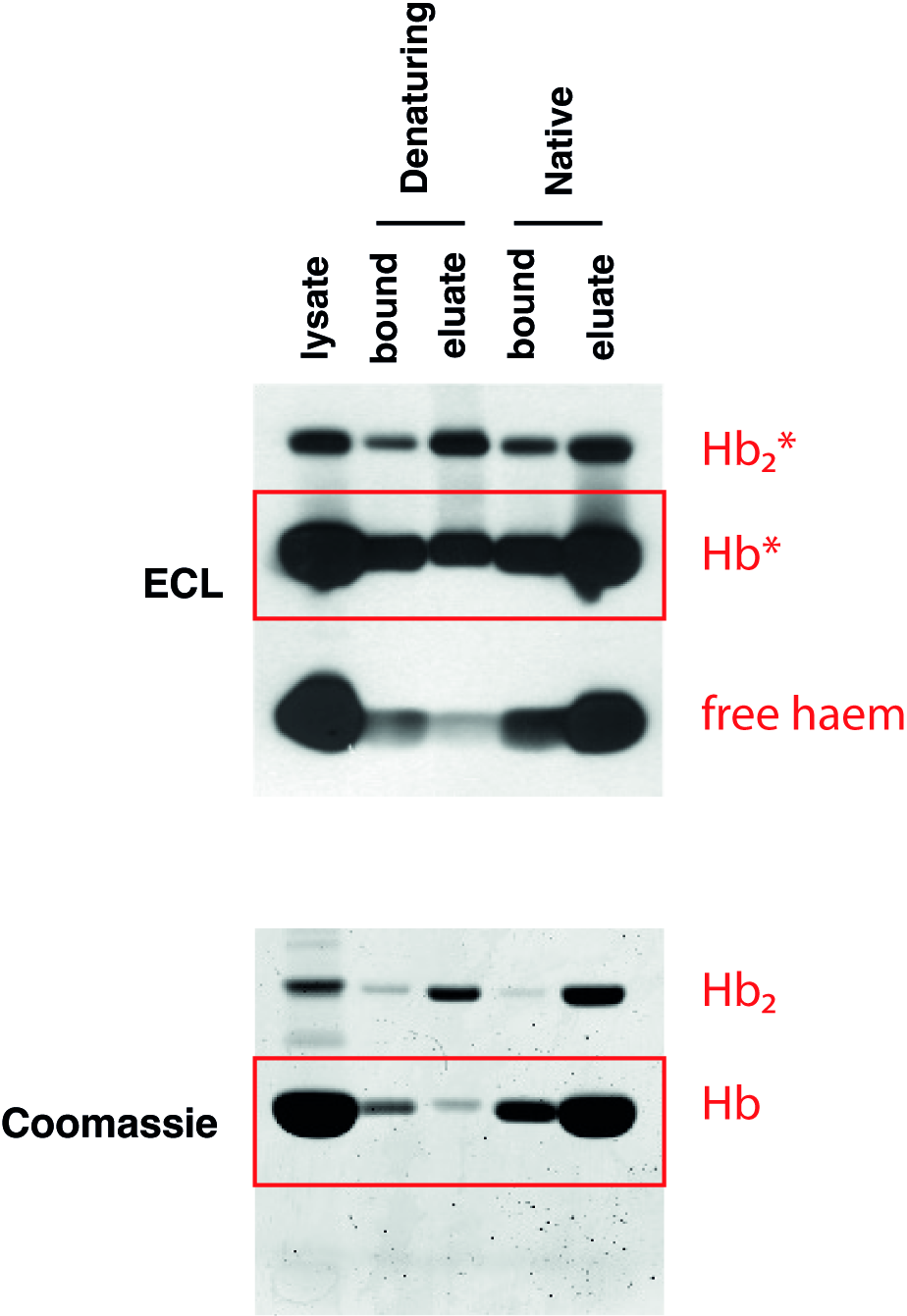

Supplement: Supplementary file 3 — Source Data for Figure 1 [file EMBJ-42-e114164-s003.zip › Figure 1/1C/ECL membrane and coomassie gel for 1C.tif]

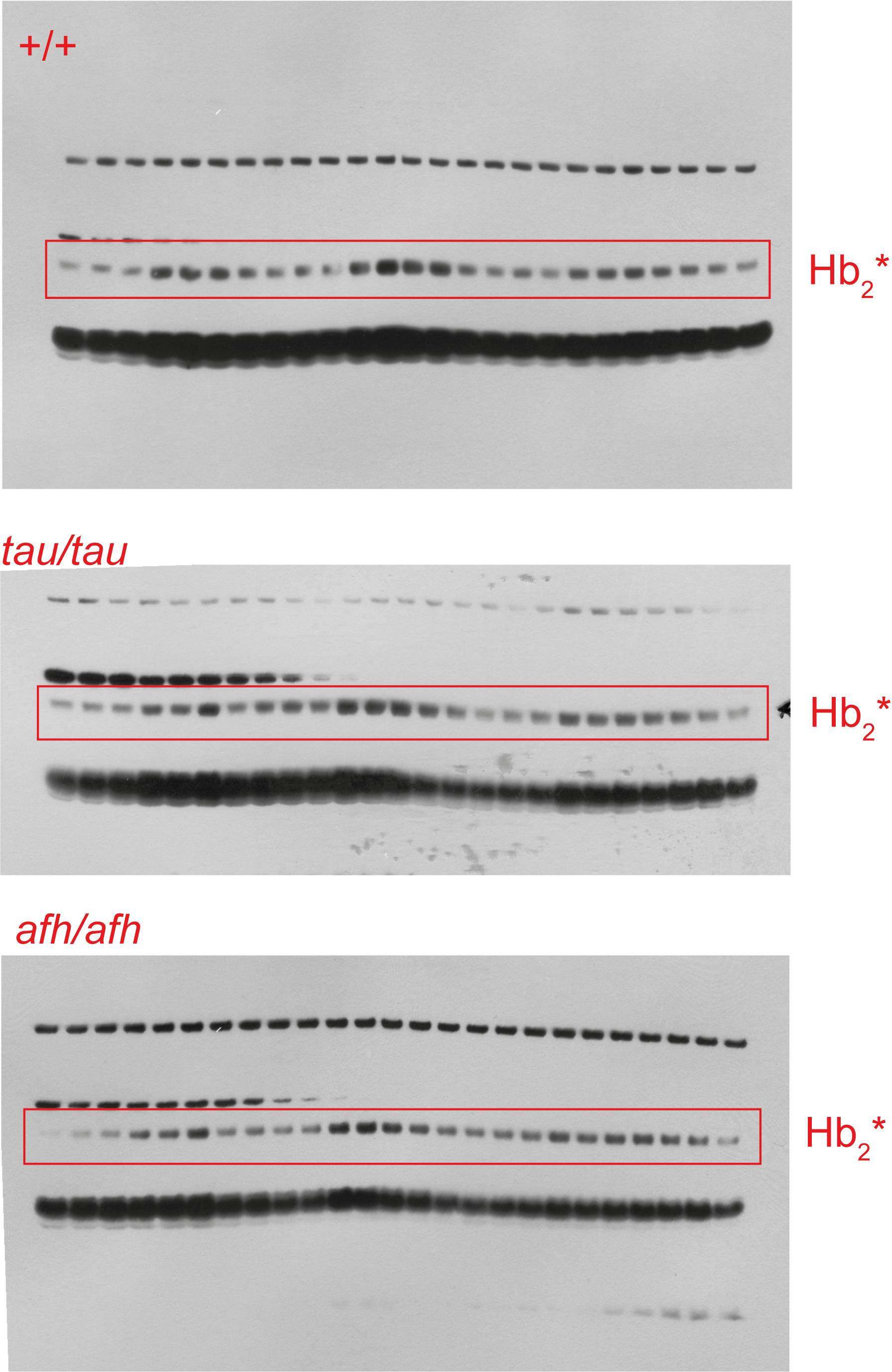

Supplement: Supplementary file 4 — Source Data for Figure 2 [file EMBJ-42-e114164-s002.zip › Figure 2/2C/ECL membrane, three genotypes.tif]

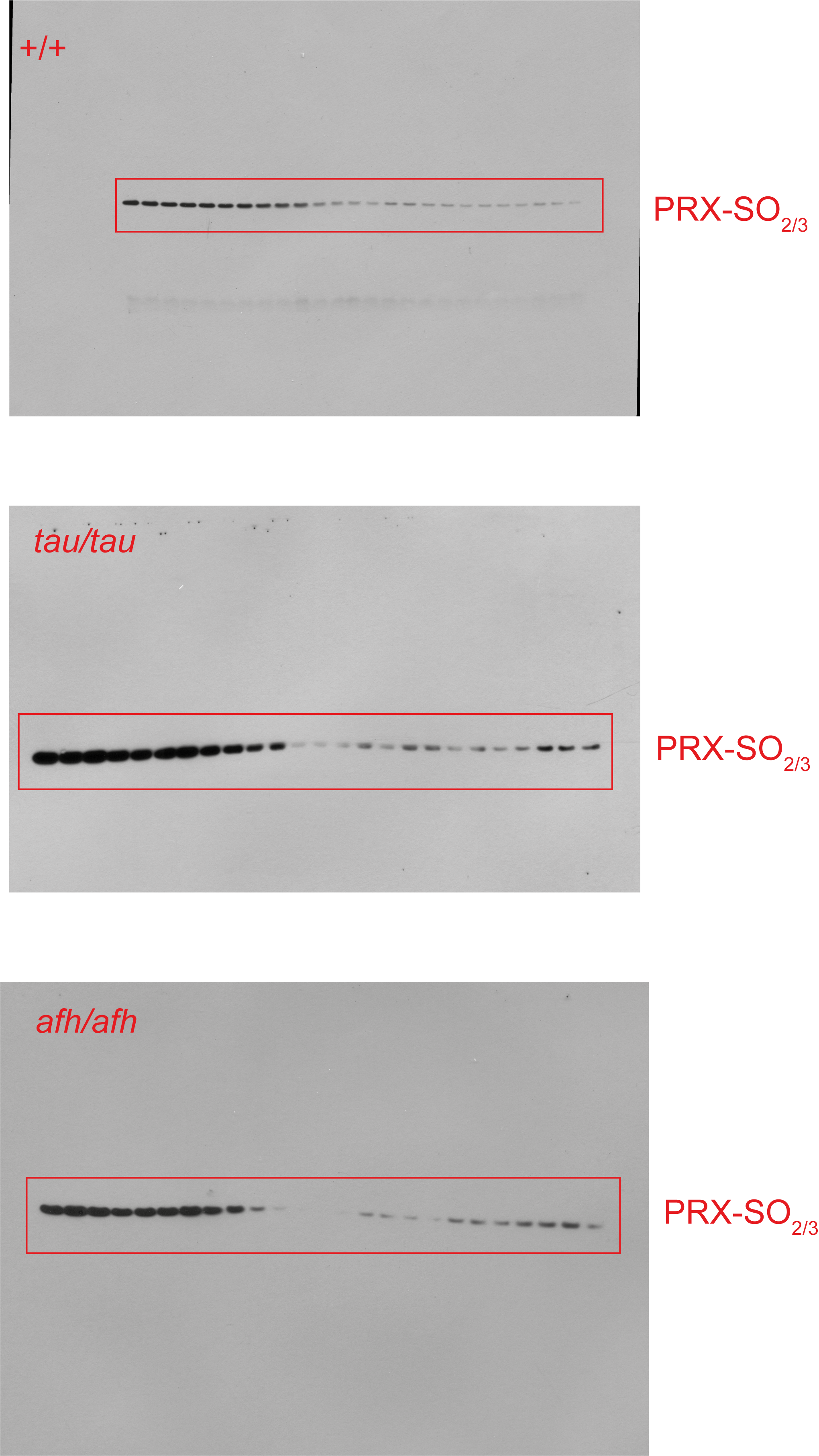

Supplement: Supplementary file 4 — Source Data for Figure 2 [file EMBJ-42-e114164-s002.zip › Figure 2/2B/PRX immunoblot, three genotypes.tif]

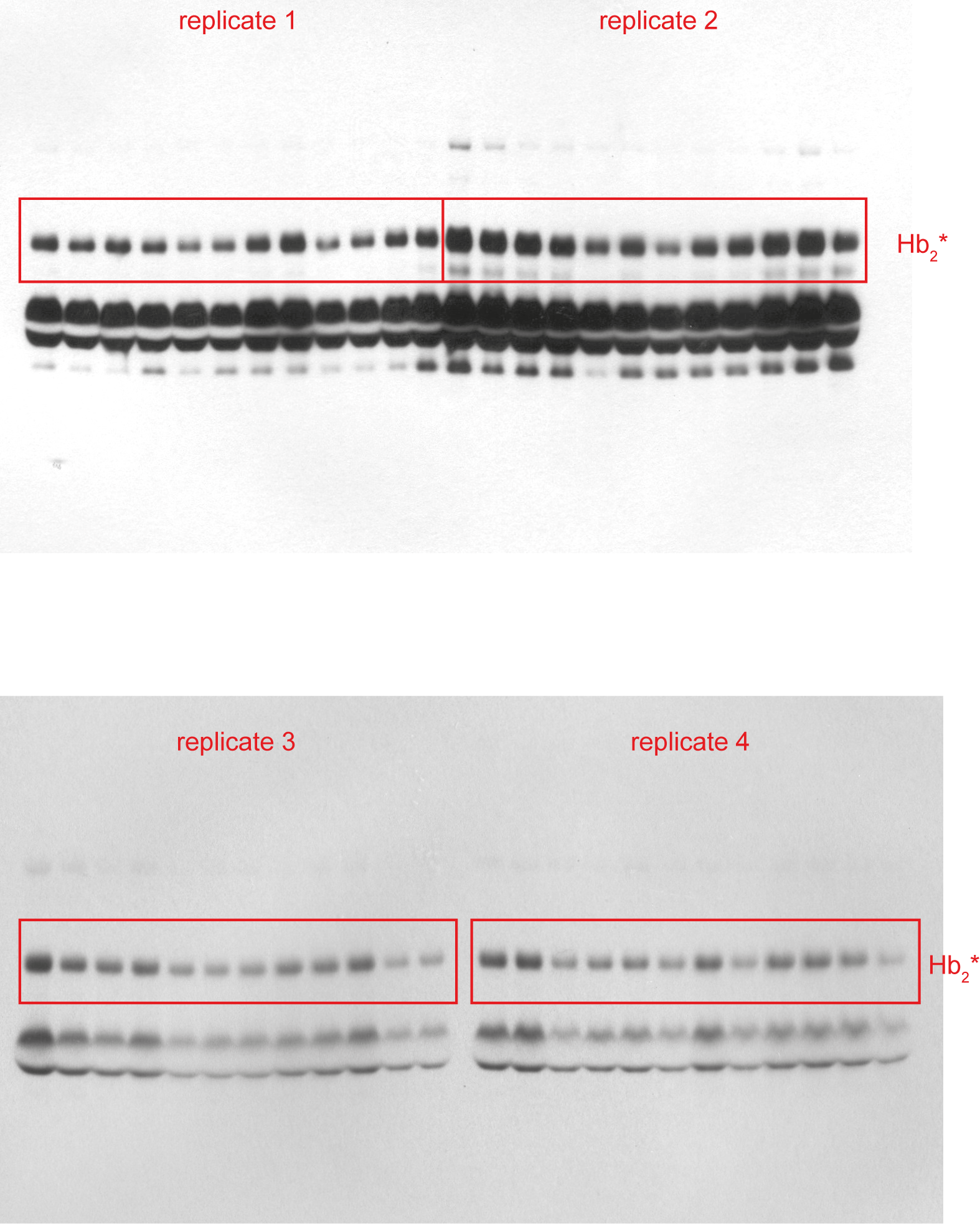

Supplement: Supplementary file 5 — Source Data for Figure 3 [file EMBJ-42-e114164-s006.zip › Figure 3/3B/ECL membranes, replicates 1-4.tif]

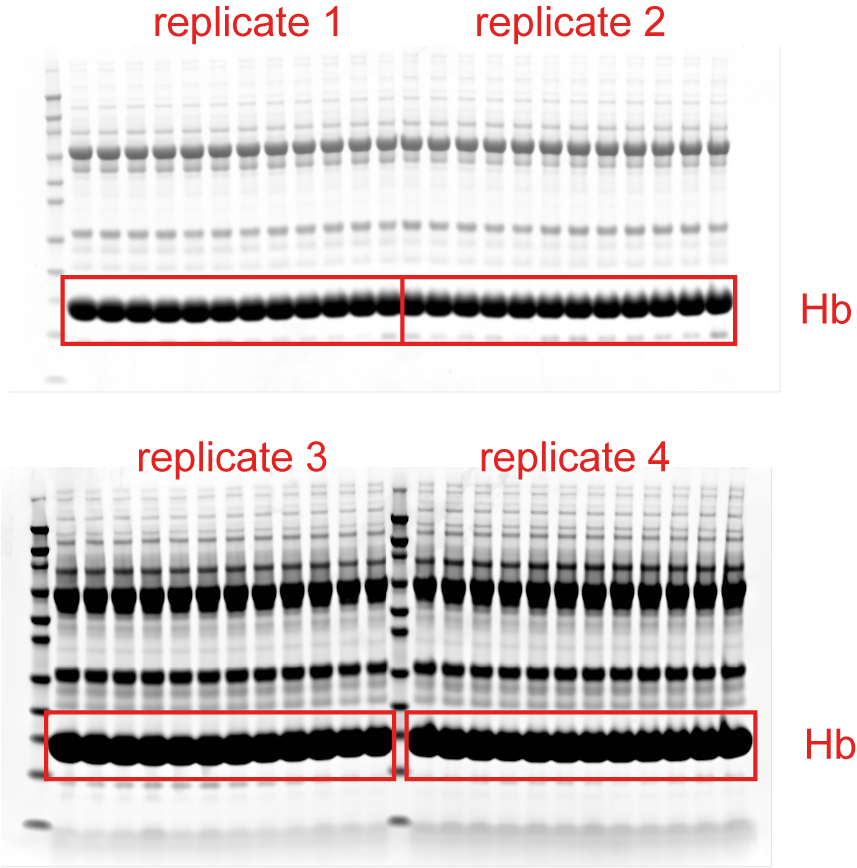

Supplement: Supplementary file 5 — Source Data for Figure 3 [file EMBJ-42-e114164-s006.zip › Figure 3/3B/coomassie gels, replicates 1-4.tif]

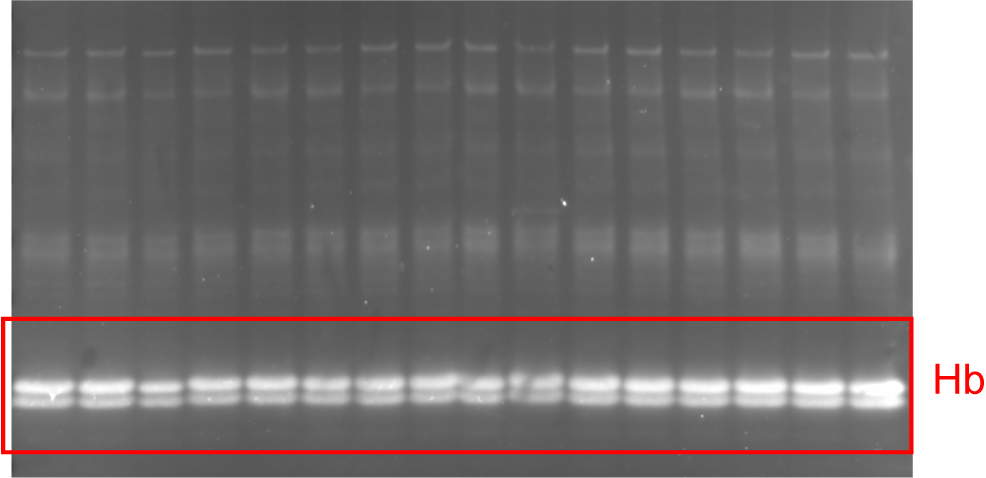

Supplement: Supplementary file 6 — Source Data for Figure 4 [file EMBJ-42-e114164-s005.zip › Figure 4/4B/SYPRO ruby gel.tif]

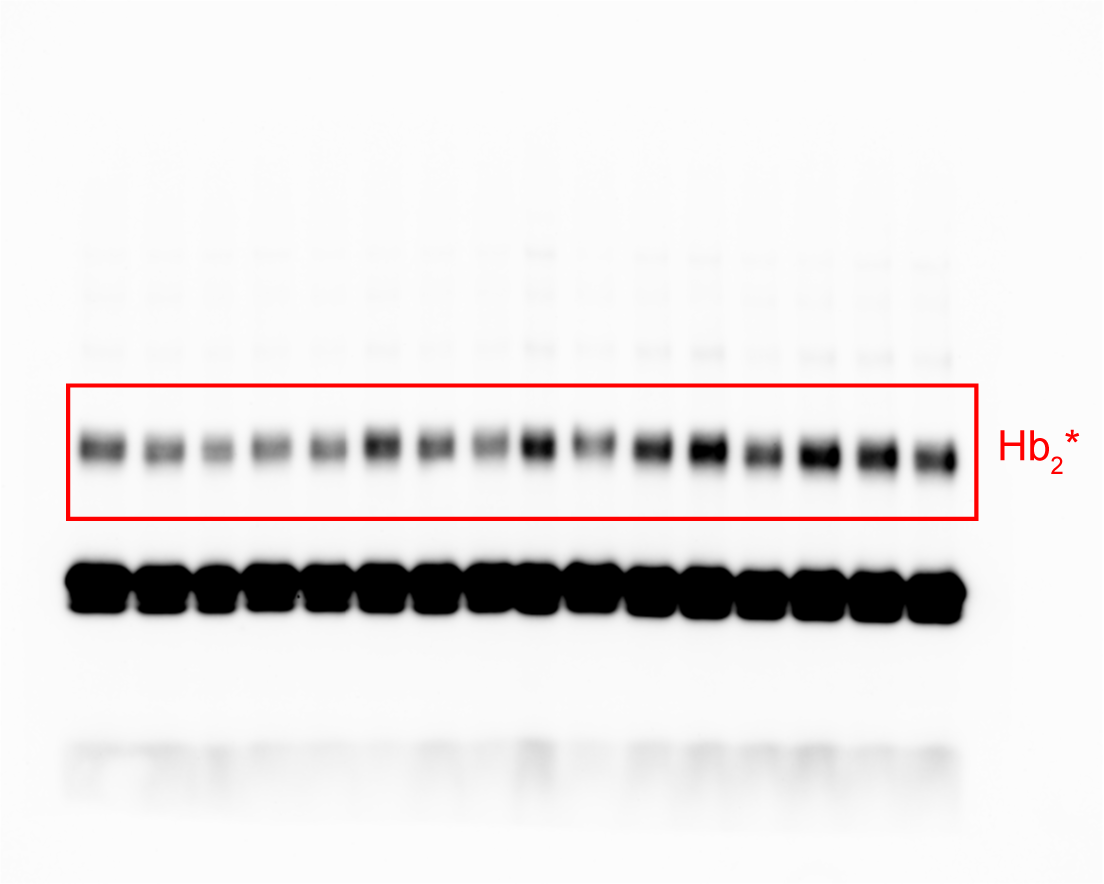

Supplement: Supplementary file 6 — Source Data for Figure 4 [file EMBJ-42-e114164-s005.zip › Figure 4/4B/ECL membrane.tif]
