# Supplementary material for: Mechanisms and physiological function of daily haemoglobin oxidation rhythms in red blood cells
Source: EMBO J. 2023 Aug 9;42(19):e114164. doi: 10.15252/embj.2023114164 (PMC10548169; doi:10.15252/embj.2023114164)
Supplement: Supplementary file 7 [file EMBJ-42-e114164-s007.pdf]

THE EMBO JOURNAL

Mechanisms and physiological function of daily haemoglobin oxidation rhythms in red blood cells

Andrew Beale, Edward Hayter, Priya Crosby, Utham Valekunja, Rachel Edgar, Johanna Chesham, Elizabeth Maywood, Fatima Labeed, Akhilesh Reddy, Kenneth Wright, Kathryn Lilley, David Bechtold, Michael Hastings, and John O'Neill
DOI: 10.15252/embj.2023114164

Corresponding author(s): John O'Neill (oneillj@mrc-lmb.cam.ac.uk) , Michael Hastings (mha@mrc-lmb.cam.ac.uk)

Review Timeline:

Transfer from Review Commons:	31st Mar 23
Editorial Decision:	25th Apr 23
Revision Received:	16th May 23
Accepted:	16th Jul 23

Editor: Karin Dumstrei

Transaction Report:

This manuscript was transferred to The EMBO JOURNAL following peer review at Review Commons.

Review #1

1. Evidence, reproducibility and clarity:

Evidence, reproducibility and clarity (Required)

Summary:

Anuculeate red blood cell (RBC) is one of the interesting biological models that indicate the presence of eukaryotic circadian system independent of transcription-translation feedback. In this manuscript, the authors set up a new method for quantifying the circadian rhythmicity in RBC. The method called "Bloody Blotting" was developed through the careful and insightful investigation of "non-specific band" observed in the western blotting of peroxiredoxin, which has been used for the circadian monitoring of RBC. The authors characterized that the "non-specific' circadian-fluctuating signals, which can be observed by ECL imaging without any antibodies(-HRP), were attributed to ferrous-haem, but not ferric-haem, cross-linked to Hb upon cell lysis. Through the Bloody Blotting, this study suggests that the circadian fluctuation of ferrous-/ferric-haem exist in human and mouse RBC, and the period of rhythmicity is not affected by the canonical clock genes.

Major comments:

1. Although the authors conducted a careful biochemical evaluation of the "Bloody Blotting" signal, it is still unclear whether the changes in the Hb* (or Hb2*) signal corresponds to the changes in the ferrous-haem level in vivo. A direct perturbation on the level of in vivo ferrous-/ferric-haem is required. For example, is the Hb* (or Hb2*) signal decreased by the administration of amyl nitrite (in mice)?
2. The authors speculated that the higher PRX-SO2/3 signal during the first 24 hrs in mice is due to the sapling time at the resting phase (line ~235). The effect of sampling time should be easily tested by maintaining the mice group in 12-hr shifted L/D cycles and sampling the blood in the same o'clock (i.e., now the active phase). This type of experiment is also critical for the evaluation of Bloody Blotting because the level of Hb*/Hb2* signals may be affected by not only the circadian timing of mice but also the daily environmental fluctuation of a biochemistry laboratory (this is particularly important for the Bloody Blotting because some of the critical steps including the cross-linking between haem and Hb are supposed to occur in a test tube). If the signal of Bloody Blotting reflects the in vivo circadian rhythmicity, the 12-hr shifted L/D mice RBCs should have 12-hr shifted Bloody Blotting fluctuation pattern.
3. Do the casein kinase inhibitors (ref: Beale, JBR 2019) affect the period of Bloody

Blotting signals?

Minor comments:

4. The authors quantify the dimer of Hb (Hb2*). This is important information but only explained in the supplementary figure legend. It should be explained in the main text. In addition, it is difficult to evaluate the fluctuation of Hb* (not Hb2*) because, as the authors stated, most of the Hb* signals are saturated. The saturation problem should be easily solved by reducing the sample loading volume. Quantification of Hb* is important at least experiments shown in figure 1A-G because the dimerization of Hb can be also affected by factors other than the in vivo ferrous-/ferric-haem conversion.
5. In the quantification of Hb2* (Figure 1A, 2E, 3C), were the signals normalized to Total Hb?
6. The explanation and interpretation of the experiment shown in figure 3D should be more careful. The pulse-oximetry was conducted in normal working day conditions (real world setting) and thus should be affected by environmental and social daily signals.
7. Typos at figure indicators in supplementary figure legends. Sup figure 1A legend refers to main figure "2" (should be 1), and figure S3 legend refers to main figure 1 (should be 3).

2. Significance:

Significance (Required)

The detection of circadian oscillation in RBC has been not easy because the experiment requires careful sample preparation and specific antibodies (Milev Methods Enzymol 2016) or a specific instrument for dielectrophesis (Henslee). The Bloody Blotting technic developed in this study will overcome this technical problem because Bloody Blotting does not rely on specific antibody and only requires conventional tools for western blotting. Because circadian biology of RBC is particularly important in the field of circadian research to evaluate the presence of eukaryotic circadian oscillator without transcription-translation feedback loops, this study will be interested a wide community of circadian clock researchers.

This reviewer has expertise in the field of circadian genomics, biochemistry, animal experiments in mice as well as human.

3. How much time do you estimate the authors will need to complete the suggested revisions:

Estimated time to Complete Revisions (Required)

(Decision Recommendation)

Between 1 and 3 months

Review #2

1. Evidence, reproducibility and clarity:

Evidence, reproducibility and clarity (Required)

Summary

The study aims to provide a new tool for detecting the hemoglobin oxidative status named "Bloody blotting". It is based on redox-sensitive covalent linkage between the haem and the haemoglobin. This linkage is a consequence of an artifactual reaction provoked by the protein extraction, due to the lysis buffer's properties.
In addition, using an in vitro (red blood cells) or in vivo (patients' blood) model the authors provide insight in the oscillating nature in the oxygen-carrying and nitrite reductase capacity of the blood, which is unaffected by the mutation of CK1εtau/tau and Fbxl3aafh/fh

Major comments:

In my honest opinion, the work does not provide interesting addition to what it is known in literature. The conclusions are summarized into a model (Fig.4) t, which is too speculative related to the amount and quality of results showed in the paper. The title is misleading. The authors did not use any mutant for clock factors, but they used a kinase (CK1εtau/tau) and a ubiquitin ligase (Fbxl3aafh/afh) mutant, which are important in the regulation of proteins belonging to the clock machinery.

Speaking of the specific points described in the paper, there are aspects that are not convincing. First, the bloody blotting is a consequence of a specific reagent contained in the lysis buffer used for the protein extraction, which reacts with the haemoglobin beta and alpha (as shown by Mass Spec). The peroxidase reaction is an artifact coming from this reaction, which simply follows the rhythmicity of peroxiding accumulation in the red blood cells, whose rhythmicity is known to be circadian. I do

not really understand the utility of this technique, which anyway is limited to the specific lysis buffer, but for scientific reasons, researchers need often a different kind of lysis buffer. This means that the approach shows strong limitation to the chemical environment of the lysis buffer. I do not see in it a useful tool that can replace antibodies.

Second, the oscillation in the peroxidase activity of PRX-SO2/3 is well known to be circadian (Edgar et al., 2012. doi:10.1038/nature11088.). Finally the circadian rhythms of red blood cells is already described and the corresponding author already published different papers about. The info provided in this paper do not add any new piece to the puzzle.

At this stage I do not consider the paper suitable for a publication.
Other observations.
Authors should describe how cells were synchronized.
In experiments performed in vitro should be used the SD instead of the SEM.

Minor comments:

There are many English mistakes in the article, also errors in naming figures in the figure legends.
Figure 1B needs an appropriate loading control.
In experiments performed in vitro should be used the SD instead of the SEM.

2. Significance:

Significance (Required)

Nature and significance of the advance
At this stage I do not see any significance or advance in the field.

Compared to existing published knowledge. The
The bloody blotting seems to be an original approach although full of limitation and based on artifactual reactions.
PRX-SO2/3 is well known to be circadian (Edgar et al., 2012. doi:10.1038/nature11088.), therefore the paper does not add any new insight.
The clock mutation do not affect the circadian rhythm in RBC is also known (O' Neill and Reddy, 2011). Therefore the results showed in the figure 3 support already published observations but do not add any particular insight.

Audience

Chronobiologists, and medical science.

Fild of expertises (reviewer)
Chronobiology, molecular biology, medical science.

Referees cross-commenting

I read your comment and they were very detailed. From my point of view I am very skeptical, as I discussed about the utility of the Bloody Blotting. Also the results showed in the paper are not very innovative fro my point of view. I would like to know what do you think about.

The rhythmicity is given by the elements present in the protein extraction. The reaction is given by the specific lysis buffer used in that experiment. Using another lysis buffer would not allow anybody to see some signal without a proper antobody. The authors claim that bloody blotting is useful because a researcher does not need to buy an antibody, but what if you don't work with a total extract of proteins? In that case, you need to change the lysis buffer, and, therefore, the bloody blotting is not useful anymore.
However, If you believe in that way, and you are two people agreeing in that, I will not oppose myself although I do not agree.

3. How much time do you estimate the authors will need to complete the suggested revisions:

Estimated time to Complete Revisions (Required)

(Decision Recommendation)

Cannot tell / Not applicable

Review #3

1. Evidence, reproducibility and clarity:

Evidence, reproducibility and clarity (Required)

Summary:

Provide a short summary of the findings and key conclusions (including methodology and model system(s) where appropriate).

The manuscript "Clock gene-independent daily regulation of haemoglobin oxidation in red blood cells" describes a new assay for quantification of haemoglobin oxidation status (bloody blotting) in anucleate red blood cells". This study furthers our understanding of the role of a post-translational oscillator (PTO) in generating circadian rhythms in biology. The authors first describe how earlier work demonstrated 24h rhythms in the intensity of chemiluminescent bands on membranes blotted with protein from red blood cells (RBCs) in the absence of antibodies after exposure to ECL. They go on to address what these bands represent (through various approaches including the use of chemical inhibitors and mass spectrometry) and conclude that they are observing haemoglobin oxidation status. It is proposed that this assay represents a novel manner (complementary to earlier work) in which to report circadian rhythms in RBCs. The manuscript goes on to demonstrate the persistence of 24h rhythms in haemoglobin oxidation status in murine RBCs, including cells isolated from two clock mutant mice. Finally, the study utilises RBCs collected from human volunteers maintained under controlled conditions and demonstrate robust rhythms via "blood blotting", this data is presented alongside pulse co-oximetry data to examine physiological relevance of these rhythms.

Major comments:

- Are the key conclusions convincing?

The key conclusions are well supported by the data. The discussion does become quite speculative, and this needs to be addressed.

- Should the authors qualify some of their claims as preliminary or speculative, or remove them altogether?

The discussion around the physiological relevance of daily regulation of haemoglobin redox status is extensive (lines 366-403) as is the discussion on RBCs and the TTFL-less clock mechanisms (lines 405-429). Whilst interesting and well thought out, and well supported by the literature, these sections are very speculative and in my opinion should be toned down.

- Would additional experiments be essential to support the claims of the paper? Request additional experiments only where necessary for the paper as it is, and do not

ask authors to open new lines of experimentation.

Further experiments would be required to support the discussion about the role of daily rhythms in haemoglobin oxidation status in regulating oxygen carrying capacity of the blood, vascular tone, body temperature and sleep-wake cycle. As the authors state, these experiments are beyond the scope of this study, but are of course of major interest. It would be more appropriate to limit the discussion to what has been demonstrated directly by the data presented, with just a few sentences speculating on physiological relevance.

- Are the suggested experiments realistic in terms of time and resources? It would help if you could add an estimated cost and time investment for substantial experiments.

If the focus of the discussion is shifted as suggested, there is no need to pursue any further experiments.

- Are the data and the methods presented in such a way that they can be reproduced?

Yes. The methods are complete, and data presented very well.

- Are the experiments adequately replicated and statistical analysis adequate?

Yes

Minor comments:

- Specific experimental issues that are easily addressable.

1.In the murine fibroblasts/RBC experiments in Figure 2 - what genotype were the wildtype controls? The main text suggests PER2::luc (line 226) but methods suggest Cry1:luc - could the authors clarify this?

2.In figure 2B and 2D the blots show two samples for each time point (except for 72h where there is just one) are these technical repeats? This should be clarified.

3.The controls for the bloody blots are referred to as Coomassie in Figure 1. In Figure 2, the controls for PRX-SO2/3 are referred to as "loading" but are Coomassie stained gels - could this be standardised? Also Figure 2D - no controls? In Figure 3B controls are referred to as 'Total Hb from Coomassie staining - I wasn't clear what this was.

4.Figure 3A "S1" and "S2" stated in legend but only "S" used in the schematic

- Are prior studies referenced appropriately?

Yes absolutely.

- Are the text and figures clear and accurate?

Mostly, few comments above.

- Do you have suggestions that would help the authors improve the presentation of their data and conclusions?

No

2. Significance:

Significance (Required)

- Describe the nature and significance of the advance (e.g. conceptual, technical, clinical) for the field.

The study describes a rapid and relatively simple assay for observing 24h rhythms in RBC function. On a technical basis - this will likely be of significant use to others in the field. Further work examining rhythms in haemoglobin oxidation in RBCs in clock mutant mice confirms independence from the transcriptional-translational feedback loop, which further supports earlier work in this field. Finally, studies in humans (bloody blotting in combination with pulse co-oximetry) provide a glimpse into the functional relevance of these daily oscillations

- Place the work in the context of the existing literature (provide references, where appropriate).

The authors have done an excellent job of reviewing the literature in the field and contextualising their data. This current data is a significant advance in the field.

- State what audience might be interested in and influenced by the reported findings.

This work will be of interest to circadian biologists and adds weight to the relatively new concept of a post-translational oscillator (PTO). Further work showing the relevance of this PTO on physiological function will be of great interest.

- Define your field of expertise with a few keywords to help the authors contextualize your point of view. Indicate if there are any parts of the paper that you do not have sufficient expertise to evaluate.

Circadian, Clock genes, mouse models,

I do not have a background in biochemistry and do not feel overly qualified to comment constructively on approaches taken to address what is driving the observed rhythmic peroxidase activity in RBCs (e.g NiNTA affinity chromatography, use of reductants to reduce thioester bonds and use of NEM to alkylate Hb cysteine residues).

Referees cross-commenting

In terms of the utility, as my review indicated, I do feel that this manuscript advances the field, providing a rapid and relatively simple way to measure rhythms in RBCs. Reviewer 1 explained this nicely in their significance summary.

3. How much time do you estimate the authors will need to complete the suggested revisions:

Estimated time to Complete Revisions (Required)

(Decision Recommendation)

Less than 1 month

Response to reviewers

We thank all reviewers for their comments and suggestions. In line, below, are our responses, marked in red. Textural changes in the manuscript are also marked in red.

Reviewer #1 (Evidence, reproducibility and clarity (Required)):

Summary:

Anucleate red blood cell (RBC) is one of the interesting biological models that indicate the presence of eukaryotic circadian system independent of transcription-translation feedback. In this manuscript, the authors set up a new method for quantifying the circadian rhythmicity in RBC. The method called "Bloody Blotting" was developed through the careful and insightful investigation of "non-specific band" observed in the western blotting of peroxiredoxin, which has been used for the circadian monitoring of RBC. The authors characterized that the "non-specific' circadian-fluctuating signals, which can be observed by ECL imaging without any antibodies(-HRP), were attributed to ferrous-haem, but not ferric-haem, cross-linked to Hb upon cell lysis. Through the Bloody Blotting, this study suggests that the circadian fluctuation of ferrous-/ferric-haem exist in human and mouse RBC, and the period of rhythmicity is not affected by the canonical clock genes.

Major comments:

1)Although the authors conducted a careful biochemical evaluation of the "Bloody Blotting" signal, it is still unclear whether the changes in the Hb* (or Hb2*) signal corresponds to the changes in the ferrous-haem level in vivo. A direct perturbation on the level of in vivo ferrous-/ferric-haem is required. For example, is the Hb* (or Hb2*) signal decreased by the administration of amyl nitrite (in mice)?

Thank you for the suggestion. We have addressed this and the second reviewer's comment in a new Figures 4 & S4 and section titled "Effect of rhythms in metHb on vascular flow and body temperature".

For clarity, we have relabelled the schematic in A to "rest phase" and "activity phase" to consolidate data from humans and mice which both feature in the manuscript. We performed two experiments to test the model in Fig 4A and perturb metHb in vivo. The first is a direct perturbation of metHb levels in vivo with sodium nitrite, an oxidising agent that causes methaemoglobinemia. Reflecting our results *ex vivo*, RBC from differentially entrained mice sampled at the same external time, but 12h apart in terms of the light:dark cycle, contained significantly different metHb levels, with more metHb in the rest phase (revised Figure 4B, C). Whereas, RBCs from mice also given nitrite in their active phase contained more metHb (and thus lower Hb_2* activity) than control (revised Figure 4B and S4). The second experiment tests the effect of sodium nitrite on core body temperature. Our hypothesis predicts that nitrite should accentuate the daytime drop in core body temperature, *via* the increased metHb-mediated production of NO to stimulate increased vasodilation (Ignacio et al 1981 and Cosby et al 2003). Revised figures 4D and E show that the effect of nitrite on body temperature (which has a large active vs inactive difference) is indeed daytime-specific.

Methods for these experiments have been added to Experimental Procedures.

2)The authors speculated that the higher PRX-SO2/3 signal during the first 24 hrs in mice is due to the sapling time at the resting phase (line ~235). The effect of sampling time should be easily tested by maintaining the mice group in 12-hr shifted L/D cycles and sampling the blood in the same o'clock (i.e., now the active phase). This type of experiment is also critical for the evaluation of Bloody Blotting because the level of Hb*/Hb2* signals may be affected by not only the circadian timing of mice but also the daily environmental fluctuation of a biochemistry laboratory (this is particularly important for the Bloody Blotting because some of the critical steps including the cross-linking between haem and Hb are supposed to occur in a test tube). If the signal of Bloody Blotting reflects the in vivo circadian rhythmicity, the 12-hr shifted L/D mice RBCs should have 12-hr shifted Bloody Blotting fluctuation pattern.

We acknowledge this possibility. To test this, we sampled RBCs from mice kept under DL and LD conditions, as detailed in the new sections in the Experimental Procedures, harvesting blood at the same clock time. This gave us blood from mice in the "active" phase and "rest" phase – labels as per Figure 4B. Figure 4B shows that Hb_2* signal significantly differs between mice in active and rest phases, even though these samples were collected and processed at the same external time.

Separately from Hb_2* activity, upon further reading of the literature we suspect that the higher PRX-$SO_{2/3}$ signal detected in mouse RBCs (Fig 2) compared with human may be due to blood acidification during animal sacrifice by CO_2. Additional text has been added to Supplementary Figure S3 to remark upon this, as follows:

> Interestingly, compared with human RBC time courses (Henslee et al., 2017; O'Neill and Reddy, 2011), we observed that murine PRX-$SO_{2/3}$ immunoreactivity was extremely high during the first 24 hours of each 72-hour time course (Figure S3A). We attribute this to the different conditions under which blood was collected: blood was collected from mice culled by CO_2 asphyxiation during their habitual rest phase by cardiac puncture and exposed immediately to atmospheric oxygen levels, whereas human blood was collected from subjects during their habitual active phase through venous collection into a vacuum-sealed collection vial. Thus, the initial high PRX-$SO_{2/3}$ signal in mice may be related to CO_2-acidification of the blood during culling, which affects PRX-$SO_{2/3}$ but does not affect Hb oxidation status.

3)Do the casein kinase inhibitors (ref: Beale, JBR 2019) affect the period of Bloody Blotting signals?

We have not experimentally addressed this as we consider it beyond the scope of the current study, which has instead focused on the in vivo relevance of the rhythms in metHb. Nevertheless, given the identical periodicity of PRX rhythms and Hb* rhythms (this paper), and the periodicity of PRX rhythms and rhythms in membrane conductance (Henslee et al, Nat Commun, 2018), we see no reason why the period lengthening of rhythms in membrane conductance reported in Beale et al, JBR, 2019 would not also been seen in PRX or Hb* rhythms.

Minor comments:

4)The authors quantify the dimer of Hb (Hb2*). This is important information but only explained in the supplementary figure legend. It should be explained in the main text. In addition, it is difficult to evaluate the fluctuation of Hb* (not Hb2*) because, as the authors stated, most of the Hb* signals are saturated. The saturation problem should be easily solved by reducing the sample loading volume. Quantification of Hb* is important at least experiments shown in figure 1A-G because the dimerization of Hb can be also affected by factors other than the in vivo ferrous-/ferric-haem conversion.

Thank you for pointing this out. Indeed the data throughout the original manuscript is Hb_2*. We have brought this explanation into Figure 1 legend and labelled all figures consistently with Hb_2*. We include quantification of Hb* and Hb_2* of the in vivo metHb perturbation experiment (Figure 4) in the uncropped membranes shown in Supplementary Figure 4. The quantification of Hb* (Supplementary Figure 4D) gives the same result as the quantification of Hb_2* (Figure 4B).

5)In the quantification of Hb2* (Figure 1A, 2E, 3C), were the signals normalized to Total Hb?

In the quantification of Hb2* throughout, signals were normalised to total protein through coomassie stain, apart from Figure 4B which used SYPRO Ruby. Each figure presents the Hb band of coomassie or SYPRO Ruby for simplicity, but the full gels are included in Supplementary Figures 1, 3 and 4.

6)The explanation and interpretation of the experiment shown in figure 3D should be more careful. The pulse-oximetry was conducted in normal working day conditions (real world setting) and thus should be affected by environmental and social daily signals.

We have changed the section to the following (edits in red):

"Remarkably, in contrast to total Hb (SpHb) that displayed no significant 24h variation, the proportion of metHb (SpMet) in the blood exhibited a striking daily variation that rose during the evening and peaked during the night (Figure 3D). These subjects were in a real-world setting, and thus affected by environmental and social cues from a normal working day. However, the evening rise and night-time peak is consistent with the reduction in Hb_2* activity at the end of the waking period in laboratory conditions (Figure 3B)."

7)Typos at figure indicators in supplementary figure legends. Sup figure 1A legend refers to main figure "2" (should be 1), and figure S3 legend refers to main figure 1 (should be 3).

Thank you for pointing this out. We have corrected these legends.

Reviewer #1 (Significance (Required)):

The detection of circadian oscillation in RBC has been not easy because the experiment requires careful sample preparation and specific antibodies (Milev Methods Enzymol 2016) or a specific instrument for dielectrophesis (Henslee). The Bloody Blotting technic developed in this study will overcome this technical problem because Bloody Blotting does not rely on specific antibody and only requires conventional tools for western blotting. Because circadian biology of RBC is particularly important in the field of circadian research to evaluate the presence of eukaryotic circadian oscillator without transcription-translation feedback loops, this study will be interested a wide community of circadian clock researchers. This reviewer has expertise in the field of circadian genomics, biochemistry, animal experiments in mice as well as human.

Thank you for taking the time to read and constructively comment on our work

Reviewer #2 (Evidence, reproducibility and clarity (Required)): **Summary**

The study aims to provide a new tool for detecting the hemoglobin oxidative status named "Bloody blotting". It is based on redox- sensitive covalent linkage between the haem and the haemoglobin. This linkage is a consequence of an artifactual reaction provoked by the protein extraction, due to the lysis buffer's properties.
In addition, using an in vitro (red blood cells) or in vivo (patients' blood) model the authors provide insight in the oscillating nature in the oxygen-carrying and nitrite reductase capacity of the blood, which is unaffected by the mutation of CK1εtau/tau and Fbxl3aafh/fh

Major comments:

In my honest opinion, the work does not provide interesting addition to what it is known in literature. The conclusions are summarized into a model (Fig.4) t, which is too speculative related to the amount and quality of results showed in the paper.

We are disappointed by the reviewer's response. The physiological basis for daily rhythms in body temperature cooling is not currently understood, this work provides a testable basis for understanding it. Whilst we understand that the reviewer might not find immediate value in the biochemical mechanisms that initially informed our investigation, the recent publication of our investigation of human brain temperature rhythms (Rzechorzek et al., Brain, 2022) demonstrates that daily biological temperature rhythms are of broad interest (Altmetric score >2000). Daily temperature rhythms have almost exclusively been assumed to result from daily rhythms in heat production, yet the evidence for a contribution via daily rhythms of cooling is equally strong yet has received scant attention.

The speculative model that the reviewer refers to was a hypothesis that drew together multiple lines of published evidence for future experimental testing, not a conclusion, and was labelled as such in the original manuscript. To accommodate the reviewer's critique, however, we tested the model with new experiments, that are included in the revised Figure 4 and section titled “Effect of rhythms in metHb on vascular flow and body temperature”.

For clarity, we have relabelled the schematic in A to “rest phase” and “active phase” to consolidate data from humans and mice which both feature in the manuscript, and described it as a hypothesis to avoid confusion. We performed two experiments to test this model. The first is a direct perturbation of metHb levels in vivo with sodium nitrite, an oxidising agent that causes methaemoglobinemia. RBCs from mice given nitrite in their active phase contain more metHb (and thus lower Hb_2* activity) than control (Figure 4B). Reflecting our results ex vivo, RBC from mice sampled 12h apart contain significantly different metHb levels, with more metHB in the rest phase (Figure 4B, C). The second experiment tests the effect of sodium nitrite on core body temperature. Our model predicts that nitrite should further reduce core body temperature in the daytime, via the increased production of metHb (Figure 4C) and vasodilation (Ignacio et al 1981 and Cosby et al 2003). Figure 4D and E show that body temperature (which has a large active vs inactive difference) is further lowered upon nitrite treatment, and that this effect is restricted to the daytime, consistent with our hypothesis.

Methods for these experiments have been added to Experimental Procedures.

The title is misleading. The authors did not use any mutant for clock factors, but they used a kinase (CK1εtau/tau) and a ubiquitin ligase (Fbxl3aafh/afh) mutant, which are important in the regulation of proteins belonging to the clock machinery.

We respectfully disagree that the title was misleading. Mice and cultured cells/tissues that are mutant for CK1 and FBXL3 demonstrably show altered clock gene activity (See Godhino et al, Science, 2007, also Meng et al, Neuron, 2008, also Fig 2). Moreover, CK1 and FBXL3 are generally regarded as key components of the circadian clock due to their critical function in the regulation of clock proteins (e.g., Hirano et al, Nat. Struct. Mol. Biol., 2016). Being anucleate, RBCs lack the capacity for changes in clock gene activity and the period of oscillation is not affected by mutations that affect the activity and period of clock gene-oscillations in nucleated cells and whole mice. Since the rhythms of Hb oxidation

persist in isolated RBCs, they cannot be dependent on clock gene activity and so must be considered to function independent of clock genes.

In light of the new data on mouse body temperature presented in revised Fig 4D/E, however, we have changed the title to better communicate the revised scope of the manuscript, as follows:

"Mechanisms and physiological function of daily haemoglobin oxidation rhythms in red blood cells"

Speaking of the specific points described in the paper, there are aspects that are not convincing. First, the bloody blotting is a consequence of a specific reagent contained in the lysis buffer used for the protein extraction, which reacts with the haemoglobin beta and alpha (as shown by Mass Spec). The peroxidase reaction is an artifact coming from this reaction, which simply follows the rhythmicity of peroxiding accumulation in the red blood cells, whose rhythmicity is known to be circadian. I do not really understand the utility of this technique, which anyway is limited to the specific lysis buffer, but for scientific reasons, researchers need often a different kind of lysis buffer. This means that the approach shows strong limitation to the chemical environment of the lysis buffer. I do not see in it a useful tool that can replace antibodies.

Apologies, we have not been clear enough. The bloody blotting is indeed a consequence of lysis, since that lysis condition fixes the cellular state at the time of lysis. In this case, the variation in Hb oxidation status is fixed at the time of lysis. The peroxidase activity we report is indeed revealed on membranes by the covalent interaction of the haem and Hb, which occurs at the point of lysis, and reports the oxidation state of the haem at the point of lysis. As we detail, haem exhibits peroxidase activity, so the signal we observe at molecular weights corresponding to Hb and Hb_2 is peroroxidase activity due to covalently bound haem, where the peroxidase activity varies with the oxidation state of the haem. We have reorganised text associated with Figure 1, including changes to the final paragraph of the section to make explicitly clear that that the rhythm is due to a fixing of the redox state of Hb at the time of lysis – that a true underlying rhythm is revealed.

This technique is indeed limited to the observation of haem-peroxidase activity in RBCs on membranes. But as we explain in the manuscript, this is a far quicker and simpler method of observing RBC circadian rhythms than other methods, including immunoblotting for peroxiredoxins. Furthermore, it is common to change lysis buffer according to the downstream purpose.

Second, the oscillation in the peroxidase activity of PRX-SO2/3 is well known to be circadian (Edgar et al., 2012. doi:10.1038/nature11088.).

Many apologies, we do not understand the point. It is indeed correct that PRX-$SO_{2/3}$ abundance oscillations have been reported in RBCs and other cells and organisms. Here we report another rhythm, separate to PRX: the rhythm in Hb:metHb. The PRX-$SO_{2/3}$ blots serve as a positive control for rhythmicity.

Finally the circadian rhythms of red blood cells is already described and the corresponding author already published different papers about. The info provided in this paper do not add any new piece to the puzzle.

Respectfully, we report a novel rhythm in RBCs and demonstrate its functional relevance in vivo in humans (Figure 3) and mice (Figure 4), i.e., it is the identity of the rhythmic species that is novel, not that there are rhythms. What we further add with this study is that rhythms are not influenced by the cellular/organismal environment during RBC development (Figure 2), occur in vivo, in freely moving people (Figure 3) and metHb has a functionally significant role in body temperature rhythms (Figure 4). Furthermore, we report a novel technique for uncovering this rhythm in RBCs.

At this stage I do not consider the paper suitable for a publication.
Other observations.
Authors should describe how cells were synchronized.

RBCs in vitro were not synchronised by external cues. As reported in the Methods section, they were maintained at constant temperature after isolation. Fibroblasts were synchronised by temperature cycles as detailed and employed previously.

In experiments performed in vitro should be used the SD instead of the SEM.

We respectfully disagree. The SEM quantifies how precisely you know the true mean of the population - in each case we use it, we also present replicates' data from which the mean is calculated (e.g. Fig 1A, Fig 2E, Fig 3B and Fig 4B). This gives the real scatter of the data, as a SD would.

Minor comments:

There are many English mistakes in the article, also errors in naming figures in the figure legends.

We have carefully re-examined the manuscript to find and fix these errors.

Figure 1B needs an appropriate loading control.

We have added the coomassie loading control to revised Figure 1B, with uncropped membranes shown in revised Supplementary Figure 1B

In experiments performed in vitro should be used the SD instead of the SEM.

SD vs SEM, see reply above.

Reviewer #2 (Significance (Required)):

Nature and significance of the advance
At this stage I do not see any significance or advance in the field.

Compared to existing published knowledge. The
The bloody blotting seems to be an original approach although full of limitation and based on artifactual reactions.
PRX-SO2/3 is well known to be circadian (Edgar et al., 2012. doi:10.1038/nature11088.), therefore the paper does not add any new insight.
The clock mutation do not affect the circadian rhythm in RBC is also known (O' Neill and Reddy, 2011). Therefore the results showed in the figure 3 support already published observations but do not add any particular insight.

It is unclear to us how the reviewer has misunderstood the scope and focus of the manuscript to such an extent. All previous work in this area by our own and other labs has been appropriately acknowledged. To reiterate the novel elements of this work:

- A daily rhythm of Hb redox state in mouse and human red blood cells, in vitro and in vivo. This was speculated about in O'Neill & Reddy (Nature, 2011) but never directly tested until now.

- That clock gene mutations that post-translationally regulate circadian period in nucleated mammalian cells do not affect circadian period in anucleate mammalian cells. O'Neill & Reddy (Nature, 2011) did not show this, rather we looked at (nucleated) fibroblasts that were deficient for Cry1/2 (a transcriptional repressor).

- A novel assay for measuring mammalian RBC rhythms - nowhere is it proposed that the assay would be useful in any other context, as the reviewer seems to imply.

- A mechanistic basis for understanding how daily rhythms in cooling of body temperature might arise, a poorly studied aspect of mammalian physiology.

The elements in this work that are not completely novel are included as controls, they are not the focus of the manuscript e.g. PRX-$SO_{2/3}$ rhythms have not previously been shown under these conditions in mouse RBCs, only human, so these blots are included as a control for rhythmicity in Fig2. Similarly, the period of oscillation of a genetically-encoded Cry1:Luc reporter in mouse fibroblasts would be predicted to be longer and shorter in Fbxl3 and Ck1 mutants, respectively, but nowhere this been published so we have included it as a control.

Audience
Chronobiologists, and medical science.

Fild of expertises (reviewer)
Chronobiology, molecular biology, medical science.

Referees cross-commenting

I read your comment and they were very detailed. From my point of view I am very skeptical, as I discussed about the utility of the Bloody Blotting. Also the results showed in the paper are not very innovative fro my point of view. I would like to know what do you think about.

The rhythmicity is given by the elements present in the protein extraction. The reaction is given by the specific lysis buffer used in that experiment. Using another lysis buffer would not allow anybody to see some signal without a proper antobody. The authors claim that bloody blotting is useful because a researcher does not need to buy an antibody, but what if you don't work with a total total extract of proteins? In that case, you need to change the lysis buffer, and, therefore, the bloody blotting is not useful anymore. However, If you believe in that way, and you are two people agreeing in that, I will not oppose myself although I do not agree.

Reviewer #3 (Evidence, reproducibility and clarity (Required)):
Summary:
Provide a short summary of the findings and key conclusions (including methodology and model system(s) where appropriate).

The manuscript "Clock gene-independent daily regulation of haemoglobin oxidation in red blood cells" describes a new assay for quantification of haemoglobin oxidation status (bloody blotting) in anucleate red blood cells". This study furthers our understanding of the role of a post-translational oscillator (PTO) in generating circadian rhythms in biology. The authors first describe how earlier work demonstrated 24h rhythms in the intensity of chemiluminescent bands on membranes blotted with protein from red blood cells (RBCs) in the absence of antibodies after exposure to ECL. They go on to address what these bands represent (through various approaches including the use of chemical inhibitors and mass spectrometry) and conclude that they are observing haemoglobin oxidation status. It is proposed that this assay represents a novel manner (complementary to earlier work) in which to report circadian rhythms in RBCs. The manuscript goes on to demonstrate the persistence of 24h rhythms in haemoglobin oxidation status in murine RBCs, including cells isolated from two clock mutant mice. Finally, the study utilises RBCs collected from human volunteers maintained under controlled conditions and demonstrate robust rhythms via "blood blotting", this data is presented alongside pulse co-oximetry data to examine physiological relevance of these rhythms.

Major comments:
-Are the key conclusions convincing?

The key conclusions are well supported by the data. The discussion does become quite speculative, and this needs to be addressed.

-Should the authors qualify some of their claims as preliminary or speculative, or remove them altogether?

The discussion around the physiological relevance of daily regulation of haemoglobin redox status is extensive (lines 366-403) as is the discussion on RBCs and the TTFL-less clock mechanisms (lines 405-429). Whilst interesting and well thought out, and well supported by the literature, these sections are very speculative and in my opinion should be toned down.

Thank you to the reviewer for both the compliment and suggestion. Indeed, these discussion sections were too long. We have reorganised the physiological relevance section to reduce its length and better accommodate the new data presented in the new experiments in Figure 4.

We have cut the TTFL-less section text by more than half.

-Would additional experiments be essential to support the claims of the paper? Request additional experiments only where necessary for the paper as it is, and do not ask authors to open new lines of experimentation.

Further experiments would be required to support the discussion about the role of daily rhythms in haemoglobin oxidation status in regulating oxygen carrying capacity of the blood, vascular tone, body temperature and sleep-wake cycle. As the authors state, these experiments are beyond the scope of this study, but are of course of major interest. It would be more appropriate to limit the discussion to what has been demonstrated directly by the data presented, with just a few sentences speculating on physiological relevance.

As above, we acknowledge that we were speculative in that section and we have curtailed the discussion as suggested.

-Are the suggested experiments realistic in terms of time and resources? It would help if you could add an estimated cost and time investment for substantial experiments.

If the focus of the discussion is shifted as suggested, there is no need to pursue any further experiments. -Are the data and the methods presented in such a way that they can be reproduced? Yes. The methods are complete, and data presented very well.
-Are the experiments adequately replicated and statistical analysis adequate?

Yes

Minor comments:

-Specific experimental issues that are easily addressable.

1.In the murine fibroblasts/RBC experiments in Figure 2 - what genotype were the wildtype controls? The main text suggests PER2::luc (line 226) but methods suggest Cry1:luc - could the authors clarify this?

Thank you for pointing out this mistake, corrected text to Cry1:luciferase

2.In figure 2B and 2D the blots show two samples for each time point (except for 72h where there is just one) are these technical repeats? This should be clarified.

Apologies, the labelling of this figure was not clear – for space reasons we only labelled every 2nd timepoint – the time course was 3-hourly. We have corrected the figure to label each timepoint.

3.The controls for the bloody blots are referred to as coomassie in Figure 1. In Figure 2, the controls for PRX-SO2/3 are referred to as "loading" but are coomassie stained gels - could this be standardised? Also Figure 2D - no controls? In Figure 3B controls are referred to as 'Total Hb from coomassie staining - I wasn't clear what this was.

Thank you. Throughout we have now labelled loading controls by their method (coomassie or SYPRO Ruby). Figure 2D is taken from the same gel as Figure 2B and so the same coomassie gel stain is used as a loading control. We have altered the figure legend to reflect this. Each figure presents the Hb band of coomassie or SYPRO Ruby for simplicity, but the full gels are included in Supplemetary Figures 1, 3 and 4. We have changed each figure legend to reflect: "coomassie stained gels were used as loading controls; the Hb band from the coomassie stained gel is shown".

4.Figure 3A "S1" and "S2" stated in legend but only "S" used in the schematic

Many thanks for pointing this out. We have corrected the schematic to S1 and S2.

-Are prior studies referenced appropriately? Yes absolutely.
-Are the text and figures clear and accurate? Mostly, few comments above.

-Do you have suggestions that would help the authors improve the presentation of their data and conclusions? No

Reviewer #3 (Significance (Required)):
-Describe the nature and significance of the advance (e.g. conceptual, technical, clinical) for the field.

The study describes a rapid and relatively simple assay for observing 24h rhythms in RBC function. On a technical basis - this will likely be of significant use to others in the field. Further work examining rhythms in haemoglobin oxidation in RBCs in clock mutant mice confirms independence from the transcriptional-translational feedback loop, which further supports earlier work in this field. Finally, studies in humans (bloody blotting in combination with pulse co-oximetry) provide a glimpse into the functional relevance of these daily oscillations

-Place the work in the context of the existing literature (provide references, where appropriate).

The authors have done an excellent job of reviewing the literature in the field and contextualising their data. This current data is a significant advance in the field.

-State what audience might be interested in and influenced by the reported findings.

This work will be of interest to circadian biologists and adds weight to the relatively new concept of a post-translational oscillator (PTO). Further work showing the relevance of this PTO on physiological function will be of great interest.

-Define your field of expertise with a few keywords to help the authors contextualize your point of view. Indicate if there are any parts of the paper that you do not have sufficient expertise to evaluate.

Circadian, Clock genes, mouse models,

I do not have a background in biochemistry and do not feel overly qualified to comment constructively on approaches taken to address what is driving the observed rhythmic peroxidase activity in RBCs (e.g NiNTA affinity chromatography, use of reductants to reduce thioester bonds and use of NEM to alkylate Hb cysteine residues).

Referees cross-commenting

In terms of the utility, as my review indicated, I do feel that this manuscript advances the field, providing a rapid and relatively simple way to measure rhythms in RBCs. Reviewer 1 explained this nicely in their significance summary.

1st Editorial Decision **25th Apr 2023**

Dear John,

Thank you for submitting your manuscript to The EMBO Journal from Review Commons where the review process was carried out.

Your MS has now been seen by the original referees #1 and 3 and their comments are provided below. The original referee #3 is now listed as referee #2.

As you can see below, the referees find the study improved and are overall supportive of the study. They have a few relative minor concerns remaining that I would like to ask you to address in a final revision.

Also, when your revised manuscript is resubmitted, we will do all our normal checks on the manuscript including figure checks, editorial checks and our publisher will also do their checks on it. I unfortunately can't trigger these checks at this stage as we need a word file and individual figure files to do this. Once this letter is triggered our source data coordinator will also get in touch with you regarding the source data needed. Please also see important comments below on how to prepare your revised version.

Just saying this to give you a heads up about the checks happening at the next stage. When you upload the revised version please upload a word doc file, individual figure files and a point-by-point response.

With best wishes

Karin

Karin Dumstrei, PhD
Senior Editor
The EMBO Journal

Instructions for preparing your revised manuscript:

Please make sure you upload a letter of response to the referees' comments together with the revised manuscript.

Please also check that the title and abstract of the manuscript are brief, yet explicit, even to non-specialists.

When assembling figures, please refer to our figure preparation guideline in order to ensure proper formatting and readability in print as well as on screen:
https://bit.ly/EMBOPressFigurePreparationGuideline
See also figure legend guidelines: https://www.embopress.org/page/journal/14602075/authorguide#figureformat

IMPORTANT: When you send the revision we will require
- a point-by-point response to the referees' comments, with a detailed description of the changes made (as a word file).
- a word file of the manuscript text.
- individual production quality figure files (one file per figure)
- a complete author checklist, which you can download from our author guidelines (https://www.embopress.org/page/journal/14602075/authorguide).
- Expanded View files (replacing Supplementary Information)
Please see out instructions to authors
https://www.embopress.org/page/journal/14602075/authorguide#expandedview

Please remember: Digital image enhancement is acceptable practice, as long as it accurately represents the original data and conforms to community standards. If a figure has been subjected to significant electronic manipulation, this must be noted in the figure legend or in the 'Materials and Methods' section. The editors reserve the right to request original versions of figures and the original images that were used to assemble the figure.

Further information is available in our Guide For Authors: https://www.embopress.org/page/journal/14602075/authorguide

Please click on the link below to submit the revision online:

Link Not Available

Referee #1:

The revised manuscript readily answered all major concerns raised by this reviewer, especially through the additional experiment summarized in new Figure 4. This reviewer noticed a few minor comments below, but the manuscript is now technically ready for publication.

Minor comments without affecting the scientific conclusions:
1) To be consistent with the other figures, please indicate molecular weight in the new Figure 4B.
2) Again, to be consistent with the other figures, please consider indicating the quantified value as normalized intensity "(%)" in Figure 4C.

Referee #2:

General summary

I have reviewed an earlier version of this manuscript on Review Commons. In brief, the authors describe the use of "bloody blotting" to quantify haemoglobin oxidation status in RBCs. The show that 24h rhythms in haemoglobin oxidation status are evident in mice and humans and importantly persist on a 24h schedule in mice with genetically altered clocks. This latest version of the manuscript goes on to address the importance of these rhythms on animal physiology, and suggest that redox status of haemoglobin likely has a direct impact on core body temperature via NO signalling and vasodilation.

Relating to my earlier review, the authors have addressed my comments in full, and have provided a more focused (and less speculative) discussion. I am pleased to see the addition of some in vivo data exploring the consequences of altering Hb oxidation status in mice and think this raises the significance of the study.

Major concerns

The authors have addressed my earlier comments in full. The only additional comments I have are on the new data in Figure 4. The authors use sodium nitrite (a known vasodilator) in vivo and show firstly that chronic administration (10 days) results in increased Hb2*activity in RBCs during the night. They then show that administration of sodium nitrite to mice in drinking water results in a daytime decrease in core body temperature.

1. The body temperature during sodium nitrite administration was recorded over two days - were these the first two days after switching to sodium nitrite? E.g. how soon is this effect seen?

2. Whilst the data shows that dosing mice with sodium nitrite causes increased RBC Hb2* activity and alters rhythms in body temperature - the second observation can't be directly attributed to the first. Care should be taken when describing this.

3. The authors write that their observations are consistent with the hypothesis that "...RBC metHb rhythms contribute to increased peripheral blood flow and vasodilation during the rest phase, allowing core body and brain temperature to cool and so facilitating the transition to sleep". It would be more accurate and reflective of the physiological parameters measured to say ...during the rest phase, this would allow core body and brain temperature to cool and so facilitate the transition to sleep".

4. If RBCs from CK1e tau/tau and FBXL3 afh/afh mice show a similar circadian period in redox state to wildtype mice, yet the animals show shorter or longer rest-activity cycles in constant conditions - what are the authors predictions for daily rhythms in body temperature in these animals?

Minor comments
Introduction - lines 52-53: word missing? "can be affected (by?) the mutations in a number..."

***
Rev_Com_number: RC-2021-01101

New_manu_number: EMBOJ-2023-114164
Corr_author: O'Neill
Title: Mechanisms and physiological function of daily haemoglobin oxidation rhythms in red blood cells

Response to reviewers
We thank the reviewers for their comments and suggestions on our revised manuscript. In line, below, are our responses, marked in red. Textural changes in the manuscript are also marked in quotation marks.

Referee #1:

The revised manuscript readily answered all major concerns raised by this reviewer, especially through the additional experiment summarized in new Figure 4. This reviewer noticed a few minor comments below, but the manuscript is now technically ready for publication.

Minor comments without affecting the scientific conclusions:
1) To be consistent with the other figures, please indicate molecular weight in the new Figure 4B.

We have now included this in the figure.

2) Again, to be consistent with the other figures, please consider indicating the quantified value as normalized intensity "(%)" in Figure 4C.

Figure 4C y-axis is now, "Normalized Hb_2* (% min-max)"

Referee #2:

General summary

I have reviewed an earlier version of this manuscript on Review Commons. In brief, the authors describe the use of "bloody blotting" to quantify haemoglobin oxidation status in RBCs. The show that 24h rhythms in haemoglobin oxidation status are evident in mice and humans and importantly persist on a 24h schedule in mice with genetically altered clocks. This latest version of the manuscript goes on to address the importance of these rhythms on animal physiology, and suggest that redox status of haemoglobin likely has a direct impact on core body temperature via NO signalling and vasodilation.

Relating to my earlier review, the authors have addressed my comments in full, and have provided a more focused (and less speculative) discussion. I am pleased to see the addition of some in vivo data exploring the consequences of altering Hb oxidation status in mice and think this raises the significance of the study.

Major concerns

The authors have addressed my earlier comments in full. The only additional comments I have are on the new data in Figure 4. The authors use sodium nitrite (a known vasodilator)

in vivo and show firstly that chronic administration (10 days) results in increased Hb2*activity in RBCs during the night. They then show that administration of sodium nitrite to mice in drinking water results in a daytime decrease in core body temperature.

1. The body temperature during sodium nitrite administration was recorded over two days - were these the first two days after switching to sodium nitrite? E.g. how soon is this effect seen?

Sodium nitrite was given for two days only; recording took place over these two days. We have updated the methods text to make this more clear. The dose in the body temperature recording experiment was higher than the chronic administration experiment for Hb_2* measurement, hence the shorter timescale.

2. Whilst the data shows that dosing mice with sodium nitrite causes increased RBC Hb2* activity and alters rhythms in body temperature - the second observation can't be directly attributed to the first. Care should be taken when describing this.

We agree with the reviewer. Our data is consistent with the hypothesis in Figure 4A but we have taken care to clarify statements that relate manipulations of Hb_2* with body temperature, pointing to established literature. We have further qualified statements, such as in the modified final paragraph of the results: "Finally, we show that the redox status of Hb may have a direct impact on core body temperature via NO-signalling and vasodilation" and in the wording below it, to reduce the suggested strength of the direct link.

3. The authors write that their observations are consistent with the hypothesis that "...RBC metHb rhythms contribute to increased peripheral blood flow and vasodilation during the rest phase, allowing core body and brain temperature to cool and so facilitating the transition to sleep". It would be more accurate and reflective of the physiological parameters measured to say ...during the rest phase, this would allow core body and brain temperature to cool and so facilitate the transition to sleep".

Thank you for the suggestion, we have changed that part of text to the following wording: "RBC metHb rhythms contribute to increased peripheral blood flow and vasodilation during the rest phase, which would allow core body and brain temperature to cool and so facilitate the transition to sleep."

4. If RBCs from CK1e tau/tau and FBXL3 afh/afh mice show a similar circadian period in redox state to wildtype mice, yet the animals show shorter or longer rest-activity cycles in constant conditions - what are the authors predictions for daily rhythms in body temperature in these animals?

This is an interesting question. The cell autonomous and tau/tau- and afh/afh-independent oscillations in RBCs should continue to contribute to cooling under constant dark conditions in tau/tau- and afh/afh-mutant animals. In these mice, daily rhythms in heat generation due to activity will also persist but with a different free running period to that of the clock in their respective RBCs that is observed *ex vivo*. Comparing these two facts alone, we might

expect the amplitude of the body temperature rhythm to be reduced with respect to wild type due to destructive interference between RBC clock-mediated cooling and activity-driven rhythms of thermogenesis. However, many aspects of physiology free-run under constant dark conditions, such as growth factor signalling due to feed-fast cycles imposed by rest-activity. Moreover, daily temperature cycles are sufficient to entrain RBCs ex vivo (O'Neill *et al.*, Nature, 2011) . With respect to individual cells therefore, constant conditions are not present, daily thermogenesis rhythms very likely entrain RBC rhythms in vivo to oscillate with a period that matches that of the whole organism, as in other peripheral cell types (see work by the Loudon and Hastings labs). Therefore, the amplitude of the body temperature rhythm would likely remain unaltered in the mutant, because it is entrained by rest-activity rhythms, albeit with a slightly altered waveform because of small changes in phase angle between the RBC clock and rest-activity cycles.

To communicate this succinctly, we have added the following to the discussion: "Indeed, altered rhythms of body temperature in tau mutant hamsters are consistent with the predicted contribution of cell autonomous metHb rhythms to cooling (Refinetti & Menaker, 1992b)."

Minor comments
Introduction - lines 52-53: word missing? "can be affected (by?) the mutations in a number..."

Thank you for spotting this mistake, we have corrected the wording to "can be affected by mutations in a number…"

1st Revision - Editorial Decision 16th Jul 2023

Dear John,

Thank you for submitting your revised manuscript to The EMBO Journal. I have now had a chance to take a look at it and all looks good.

I am therefore very pleased to accept the manuscript for publication here.

Congratulations on a nice study!

With best wishes

Karin

Karin Dumstrei, PhD
Senior Editor
The EMBO Journal

Please note that it is EMBO Journal policy for the transcript of the editorial process (containing referee reports and your response letter) to be published as an online supplement to each paper. If you do NOT want this, you will need to inform the Editorial Office via email immediately. More information is available here:
https://emboj.embopress.org/about#Transparent_Process

Your manuscript will be processed for publication in the journal by EMBO Press. Manuscripts in the PDF and electronic editions of The EMBO Journal will be copy edited, and you will be provided with page proofs prior to publication. Please note that supplementary information is not included in the proofs.

Please note that you will be contacted by Wiley Author Services to complete licensing and payment information. The 'Page Charges Authorization Form' is available here: https://www.embopress.org/pb-assets/embo-site/tej_apc.pdf

EMBO Press participates in many Publish and Read agreements that allow authors to publish Open Access with reduced/no publication charges. Check your eligibility: https://authorservices.wiley.com/author-resources/Journal-Authors/open-access/affiliation-policies-payments/index.html

Should you be planning a Press Release on your article, please get in contact with embojournal@wiley.com as early as possible, in order to coordinate publication and release dates.

If you have any questions, please do not hesitate to call or email the Editorial Office. Thank you for your contribution to The EMBO Journal.

***
Rev_Com_number: RC-2021-01101
New_manu_number: EMBOJ-2023-114164R
Corr_author: O'Neill
Title: Mechanisms and physiological function of daily haemoglobin oxidation rhythms in red blood cells

EMBO Press Author Checklist

Corresponding Author Name: John O'Neill	
Journal Submitted to: EMBO J	
Manuscript Number: EMBOJ-2023-114164	

USEFUL LINKS FOR COMPLETING THIS FORM
The EMBO Journal - Author Guidelines
EMBO Reports - Author Guidelines
Molecular Systems Biology - Author Guidelines
EMBO Molecular Medicine - Author Guidelines

Reporting Checklist for Life Science Articles (updated January

This checklist is adapted from Materials Design Analysis Reporting (MDAR) Checklist for Authors. MDAR establishes a minimum set of requirements in transparent reporting in the life sciences (see Statement of Task: 10.31222/osf.io/9sm4x). Please follow the journal's guidelines in preparing your manuscript.

Please note that a copy of this checklist will be published alongside your article.

Abridged guidelines for figures

1. Data

The data shown in figures should satisfy the following conditions:

- the data were obtained and processed according to the field's best practice and are presented to reflect the results of the experiments in an accurate and unbiased manner.
- ideally, figure panels should include only measurements that are directly comparable to each other and obtained with the same assay.
- plots include clearly labeled error bars for independent experiments and sample sizes. Unless justified, error bars should not be shown for technical
- if n<5, the individual data points from each experiment should be plotted. Any statistical test employed should be justified.
- Source Data should be included to report the data underlying figures according to the guidelines set out in the authorship guidelines on Data

2. Captions

Each figure caption should contain the following information, for each panel where they are relevant:

- a specification of the experimental system investigated (eg cell line, species name).
- the assay(s) and method(s) used to carry out the reported observations and measurements.
- an explicit mention of the biological and chemical entity(ies) that are being measured.
- an explicit mention of the biological and chemical entity(ies) that are altered/varied/perturbed in a controlled manner.
- the exact sample size (n) for each experimental group/condition, given as a number, not a range;
- a description of the sample collection allowing the reader to understand whether the samples represent technical or biological replicates (including how many animals, litters, cultures, etc.).
- a statement of how many times the experiment shown was independently replicated in the laboratory.
- definitions of statistical methods and measures:
  - common tests, such as t-test (please specify whether paired vs. unpaired), simple χ2 tests, Wilcoxon and Mann-Whitney tests, can be unambiguously identified by name only, but more complex techniques should be described in the methods section;
  - are tests one-sided or two-sided?
  - are there adjustments for multiple comparisons?
  - exact statistical test results, e.g., P values = x but not P values < x;
  - definition of 'center values' as median or average;
  - definition of error bars as s.d. or s.e.m.

Please complete ALL of the questions below.
Select "Not Applicable" only when the requested information is not relevant for your study.

Materials

Newly Created Materials	Information included in the manuscript?	In which section is the information available? (Reagents and Tools Table, Materials and Methods, Figures, Data Availability Section)
New materials and reagents need to be available; do any restrictions apply?	Not Applicable	

Antibodies	Information included in the manuscript?	In which section is the information available? (Reagents and Tools Table, Materials and Methods, Figures, Data Availability Section)
For **antibodies** provide the following information:<br>- Commercial antibodies: RRID (if possible) or supplier name, catalogue number and or/clone number<br>- Non-commercial: RRID or citation	Yes	Materials and Methods

DNA and RNA sequences	Information included in the manuscript?	In which section is the information available? (Reagents and Tools Table, Materials and Methods, Figures, Data Availability Section)
**Short novel DNA or RNA including primers, probes**: provide the sequences.	Not Applicable	

Cell materials	Information included in the manuscript?	In which section is the information available? (Reagents and Tools Table, Materials and Methods, Figures, Data Availability Section)
**Cell lines:** Provide species information, strain. Provide accession number in repository **OR** supplier name, catalog number, clone number, and/**OR** RRID.	Yes	Materials and Methods
**Primary cultures:** Provide species, strain, sex of origin, genetic modification status.	Yes	Materials and Methods
Report if the cell lines were recently **authenticated** (e.g., by STR profiling) and tested for mycoplasma contamination.	Yes	Materials and Methods

Experimental animals	Information included in the manuscript?	In which section is the information available? (Reagents and Tools Table, Materials and Methods, Figures, Data Availability Section)
**Laboratory animals or Model organisms:** Provide species, strain, sex, age, genetic modification status. Provide accession number in repository **OR** supplier name, catalog number, clone number, **OR** RRID.	Yes	Materials and Methods
**Animal observed in or captured from the field:** Provide species, sex, and age where possible.	Not Applicable	
Please detail **housing and husbandry conditions**.	Yes	Materials and Methods

Plants and microbes	Information included in the manuscript?	In which section is the information available? (Reagents and Tools Table, Materials and Methods, Figures, Data Availability Section)
**Plants:** provide species and strain, ecotype and cultivar where relevant, unique accession number if available, and source (including location for collected wild specimens).	Not Applicable	
**Microbes:** provide species and strain, unique accession number if available, and source.	Not Applicable	

Human research participants	Information included in the manuscript?	In which section is the information available? (Reagents and Tools Table, Materials and Methods, Figures, Data Availability Section)
If collected and within the bounds of privacy constraints report on age, sex and gender or ethnicity for all study participants.	Yes	Materials and Methods

Core facilities	Information included in the manuscript?	In which section is the information available? (Reagents and Tools Table, Materials and Methods, Figures, Data Availability Section)
If your work benefited from core facilities, was their service mentioned in the acknowledgments section?	Yes	Materials and Methods

Design

Study protocol	Information included in the manuscript?	In which section is the information available? (Reagents and Tools Table, Materials and Methods, Figures, Data Availability Section)
If study protocol has been **pre-registered, provide DOI in the manuscript**. For clinical trials, provide the trial registration number **OR** cite DOI.	Not Applicable	
Report the **clinical trial registration number** (at ClinicalTrials.gov or equivalent), where applicable.	Not Applicable	

Laboratory protocol	Information included in the manuscript?	In which section is the information available? (Reagents and Tools Table, Materials and Methods, Figures, Data Availability Section)
Provide DOI OR other citation details if **external detailed step-by-step protocols** are available.	Not Applicable	

Experimental study design and statistics	Information included in the manuscript?	In which section is the information available? (Reagents and Tools Table, Materials and Methods, Figures, Data Availability Section)
Include a statement about **sample size** estimate even if no statistical methods were used.	Yes	Materials and Methods, and Figures
Were any steps taken to minimize the effects of subjective bias when allocating animals/samples to treatment (e.g. **randomization procedure**)? If yes, have they been described?	Not Applicable	
Include a statement about **blinding** even if no blinding was done.	Not Applicable	
Describe **inclusion/exclusion criteria** if samples or animals were excluded from the analysis. Were the criteria pre-established?<br><br>If sample or data points were omitted from analysis, report if this was due to attrition or intentional exclusion and provide justification.	Not Applicable	
For every figure, are **statistical tests** justified as appropriate? Do the data meet the assumptions of the tests (e.g., normal distribution)? Describe any methods used to assess it. Is there an estimate of variation within each group of data? Is the variance similar between the groups that are being statistically compared?	Yes	Materials and Methods, and Figures

Sample definition and in-laboratory replication	Information included in the manuscript?	In which section is the information available? (Reagents and Tools Table, Materials and Methods, Figures, Data Availability Section)
In the figure legends: state number of times the experiment was **replicated** in laboratory.	Yes	Figure legends
In the figure legends: define whether data describe **technical or biological replicates**.	Yes	Figure legends

Ethics

Ethics	Information included in the manuscript?	In which section is the information available? (Reagents and Tools Table, Materials and Methods, Figures, Data Availability Section)
Studies involving **human participants**: State details of **authority granting ethics approval** (IRB or equivalent committee(s), provide reference number for approval.	Yes	Materials and Methods
Studies involving **human participants**: Include a statement confirming that **informed consent** was obtained from all subjects and that the experiments conformed to the principles set out in the WMA Declaration of Helsinki and the Department of Health and Human Services Belmont Report.	Yes	Materials and Methods
Studies involving **human participants:** For publication of **patient photos**, include a statement confirming that consent to publish was obtained.	Not Applicable	
Studies involving experimental **animals**: State details of **authority granting ethics approva**l (IRB or equivalent committee(s), provide reference number for approval. Include a statement of compliance with ethical regulations.	Yes	Materials and Methods
Studies involving **specimen and field samples:** State if relevant **permits** obtained, provide details of authority approving study; if none were required, explain why.	Not Applicable	

Dual Use Research of Concern (DURC)	Information included in the manuscript?	In which section is the information available? (Reagents and Tools Table, Materials and Methods, Figures, Data Availability Section)
Could your study fall under dual use research restrictions? Please check biosecurity documents and list of **select agents and toxins** (CDC): https://www.selectagents.gov/sat/list.htm	Not Applicable	
If you used a select agent, is the security level of the lab appropriate and reported in the manuscript?	Not Applicable	
If a study is subject to dual use research of concern regulations, is the name of the **authority granting approval and reference number** for the regulatory approval provided in the manuscript?	Not Applicable	

Reporting

The MDAR framework recommends adoption of discipline-specific guidelines, established and endorsed through community initiatives. Journals have their own policy about requiring specific guidelines and recommendations to complement MDAR.

Adherence to community standards	Information included in the manuscript?	In which section is the information available? (Reagents and Tools Table, Materials and Methods, Figures, Data Availability Section)
State if relevant guidelines or checklists (e.g., **ICMJE, MIBBI, ARRIVE, PRISMA**) have been followed or provided.	Yes	ARRIVE guidelines followed for mouse experiments in Materials and Methods
For **tumor marker prognostic studies,** we recommend that you follow the **REMARK** reporting guidelines (see link list at top right). See author guidelines, under 'Reporting Guidelines'. Please confirm you have followed these guidelines.	Not Applicable	
For **phase II and III randomized controlled trials**, please refer to the **CONSORT** flow diagram (see link list at top right) and submit the CONSORT checklist (see link list at top right) with your submission. See author guidelines, under 'Reporting Guidelines'. Please confirm you have submitted this list.	Not Applicable	

Data Availability

Data availability	Information included in the manuscript?	In which section is the information available? (Reagents and Tools Table, Materials and Methods, Figures, Data Availability Section)
Have **primary datasets** been deposited according to the journal's guidelines (see 'Data Deposition' section) and the respective accession numbers provided in the Data Availability Section?	Not Applicable	
Were **human clinical and genomic datasets** deposited in a public access-controlled repository in accordance to ethical obligations to the patients and to the applicable consent agreement?	Not Applicable	
Are **computational models** that are central and integral to a study available without restrictions in a machine-readable form? Were the relevant accession numbers or links provided?	Not Applicable	
If publicly available data were reused, provide the respective **data citations in the reference list**.	Not Applicable	
